# *2mit*, an Intronic Gene of *Drosophila melanogaster timeless2*, Is Involved in Behavioral Plasticity

**DOI:** 10.1371/journal.pone.0076351

**Published:** 2013-09-30

**Authors:** Francesca Baggio, Andrea Bozzato, Clara Benna, Emanuela Leonardi, Ottavia Romoli, Moira Cognolato, Silvio C. E. Tosatto, Rodolfo Costa, Federica Sandrelli

**Affiliations:** Dipartimento di Biologia, Università degli Studi di Padova Padova, Italy; Alexander Fleming Biomedical Sciences Research Center, Greece

## Abstract

**Background:**

Intronic genes represent ~6% of the total gene complement in *Drosophila melanogaster* and ~85% of them encode for proteins. We recently characterized the *D. melanogaster* *timeless2* (*tim2*) gene, showing its active involvement in chromosomal stability and light synchronization of the adult circadian clock. The protein coding gene named *2mit* maps on the 11^th^ *tim2* intron in the opposite transcriptional orientation.

**Methodology/Principal Findings:**

Here we report the molecular and functional characterization of *2mit*. The *2mit* gene is expressed throughout *Drosophila* development, localizing mainly in the nervous system during embryogenesis and mostly in the mushroom bodies and ellipsoid body of the central complex in the adult brain. *In* *silico* analyses revealed that *2mit* encodes a putative leucine-Rich Repeat transmembrane receptor with intrinsically disordered regions, harboring several fully conserved functional interaction motifs in the cytosolic side. Using insertional mutations, tissue-specific over-expression, and down-regulation approaches, it was found that *2mit* is implicated in adult short-term memory, assessed by a courtship conditioning assay. In *D. melanogaster*, *tim2* and *2mit* do not seem to be functionally related. Bioinformatic analyses identified 2MIT orthologs in 21 *Drosophilidae*, 4 *Lepidoptera* and in *Apis mellifera*. In addition, the *tim2-2mit* host-nested gene organization was shown to be present in *A. mellifera* and maintained among *Drosophila* species. Within the *Drosophilidae 2mit*-hosting *tim2* intron, *in* *silico* approaches detected a neuronal specific transcriptional binding site which might have contributed to preserve the specific host-nested gene association across *Drosophila* species.

**Conclusions/Significance:**

Taken together, these results indicate that *2mit*, a gene mainly expressed in the nervous system, has a role in the behavioral plasticity of the adult *Drosophila* . The presence of a putative *2mit* regulatory enhancer within the *2mit-*hosting *tim2* intron could be considered an evolutionary constraint potentially involved in maintaining the *tim2-2mit* host-nested chromosomal architecture during the evolution of *Drosophila* species.

## Introduction

Any gene whose entire coding sequence lies within the bounds of a larger external gene is defined as a nested (or embedded) gene. The most common eukaryotic nested genes are completely embedded within large introns of their hosts, frequently oriented in the opposite transcriptional direction [1,2]. Recent data suggest that nested genes are widespread among Metazoans, representing ~6% of the total gene complement in *Drosophila melanogaster*, while ~2.1 and 0.5% in *Caenorhabditis elegans* and *Homo sapiens* genomes, respectively [2,3]. In *D. melanogaster*, nearly 85% of nested genes are predicted to encode for proteins, while the remaining generate non-coding RNAs [3,4].

Several investigations have been performed in order to evaluate the potential biological or evolutionary meaning of the host-nested gene organization in eukaryotic genomes. It has been hypothesized that nesting is favored by the presence of functional and/or transcriptional regulatory interactions between nested and host members [5]. However, a recent comparative analysis of expression profiles for 109 human and 752 *D. melanogaster* host-nested pairs did not show any significant correlations [3,5]. The presence of nested gene structures in Eukaryotes is currently considered an evolutionarily neutral process in which long intronic sequences provide a niche for gene insertion [2,3]. Nevertheless, phylogenetic analyses have indicated that the nesting phenomenon was preserved in certain cases along the evolution. Two independent surveys analyzing several host-nested pairs in different *Drosophilidae* reported that only 20-34% of embedded gene relationships has been conserved in non-*melanogaster* species [1,6], suggesting that evolutionary constraints maintained the host-nested genomic architecture across the species in those cases.

In 2010, we characterized the *D. melanogaster timeless2* (*tim2* or *timeout*) locus [7], the paralog of the circadian clock component *timeless1* (*tim1* [8]). *tim2* is widely expressed during development, and in the adult brain is localized mainly in the T1 basket neurons of the optic lobes and in the central complex. *tim2* is an essential gene involved in maintaining chromosome integrity during development. Moreover, it has been implicated in light synchronization of the circadian clock in the adult fly [7].

*tim2* is a 75 kb complex locus composed of 18 exons and 17 introns, which harbors four nested transcribed sequences, *CG34308*, *BK002510*, *2mit*, and *AY118619*. Among these, only *2mit*, located on the 11^th^ intron of *tim2*, represents an embedded protein-encoding gene [7].

Here, we report the molecular and functional characterization of the *D. melanogaster 2mit* nested gene. We showed that *2mit* is actively transcribed during embryogenesis, localizing in the developing nervous system. In the adult brain its expression is localized mainly in the mushroom bodies (MBs) and ellipsoid body (EB) of the central complex. *In silico* analyses indicated that 2MIT is a Leucine-Rich Repeat (LRR) transmembrane protein. Using insertional mutations, tissue-specific over-expression and RNA interference-mediated down-regulation, we demonstrated that *2mit* is involved in adult behavioral plasticity, evaluated as short-term memory by a courtship conditioning assay. When nested *2mit* and host *tim2* gene functions were compared, no evident functional relationship became apparent. However, bioinformatic analyses identified 2MIT orthologs among 21 genome-sequenced species belonging to the *Drosophila* genus, in 4 *Lepidoptera* and in the hymenopteran *Apis mellifera*. The chromosomal *tim2* locus organization, with *2mit* embedded within a *tim2* intron, was found in *A. mellifera* and all the examined *Drosophilidae*. The possible presence of evolutionary constraints contributing to preserve the *tim2-2mit* host-nested gene association across *Drosophila* species will be discussed.

## Results

### *2mit* gene structure and protein sequence analysis

**Figure 1:**
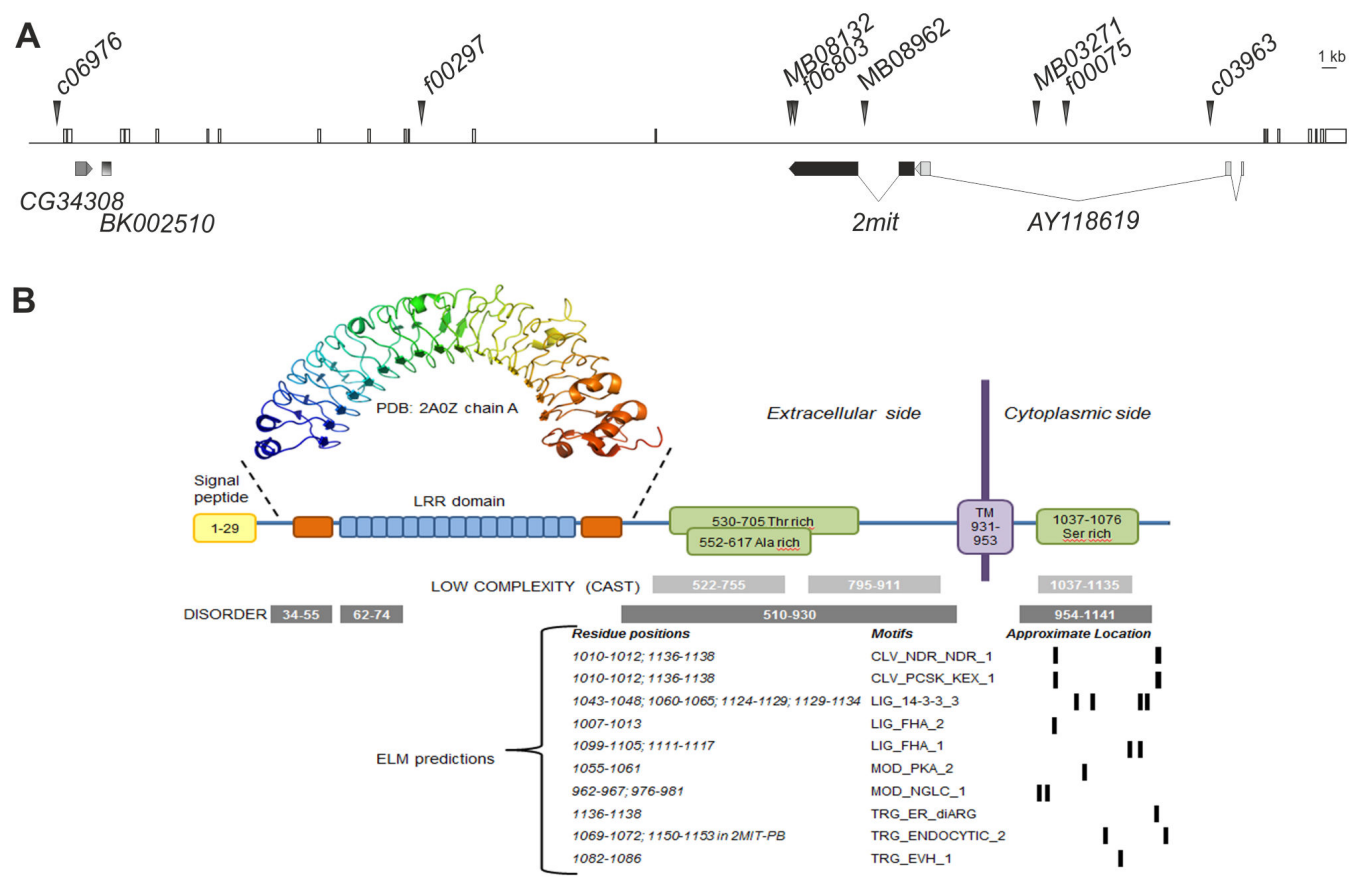
*tim2-2mit* host-nested gene organization in *D. melanogaster*. (A) Schematic representation of the *tim2* locus showing positions and intron-exon structures for the four nested sequences (*CG34308*, BK002510, *2mit* and AY118619) with only *2mit* representing a protein coding gene. Arrowheads show positions of *PB* (indicated with *c* or *f*) or *MB* transposons in different insertional strains. (B) Diagram showing the organization of protein domains contained in 2MIT. The signal peptide (yellow), the LRR domain with 16 repeat units (blue) and their N- and C- flanking regions (orange), the transmembrane region (TM, purple), and the presence of Ala-, Thr-, Ser-rich domains (green) are shown both for the extracellular and cytoplasmic portions of the protein (drawing not to scale). The 3D-structure at the top represents the 2MIT LRR repeats of the Toll-like receptor 3 crystal structure (PDB code: 2A0Z, chain A). The predictions of sequence features (low complexity and disorder) are listed at the bottom as grey and black rectangles. Linear motifs found with ELM with their approximate position are indicated by black vertical bars.

*D. melanogaster 2mit* (FBgn0260793) is organized in 2 exons and 1 intron and maps in an opposite transcriptional orientation within the 11^th^ intron of the *tim2* locus (Figure 1A [7]). *2mit* conceptual translation originates a 1141 aa protein (NP_650258) with a predicted molecular weight of ~125 kDa, a theoretical pI of 6.53 and a slightly hydrophilic Grand Average of Hydropathicity (GRAVY) value of -0.396. A transmembrane domain spanning 931-953 residues was recognized by different software tools with the N- and C-terminal regions localized in the extracellular space and cytoplasmic compartment, respectively. The N-terminal region was found to contain a signal peptide with a cleavage site at position 29. These analyses strongly suggest that the mature form of the 2MIT protein is ~122 kDa in size, 1113 aa long, and a type I single-pass transmembrane protein. The 902 aa N-terminus is suggested to be exposed to the extracellular (or luminal) space, while the 187 aa C-terminus is cytoplasmic (Figure 1B). The extracellular region is suggested to be composed of a structured domain and a long disordered region. The latter is characterized by low complexity sequences (residues 522-755 and 795-911) with a Thr-rich domain spanning 530-705 residues and a partially overlapping Ala-rich domain between 552-617 residues. The cytosolic domain is predicted to be prevalently disordered and contains a Ser-rich domain. In this region, the ELM server recognized several functional motifs, such as binding sites for 14-3-3 protein/s, Forkhead-associated (FHA) 1 and 2 factors, and a phosphorylation site for protein kinase A (PKA; Figure 1B).

FlyBase reported a second 2MIT protein isoform predicted to be 13 residues longer, possibly originating from translational stop codon read-through [9]. The C-terminal region of this longer 2MIT isoform (1155 aa) contains a TRG_endocytic motif, which is implicated in vesicular trafficking of different molecules (Figure 1B).

**Figure 2:**
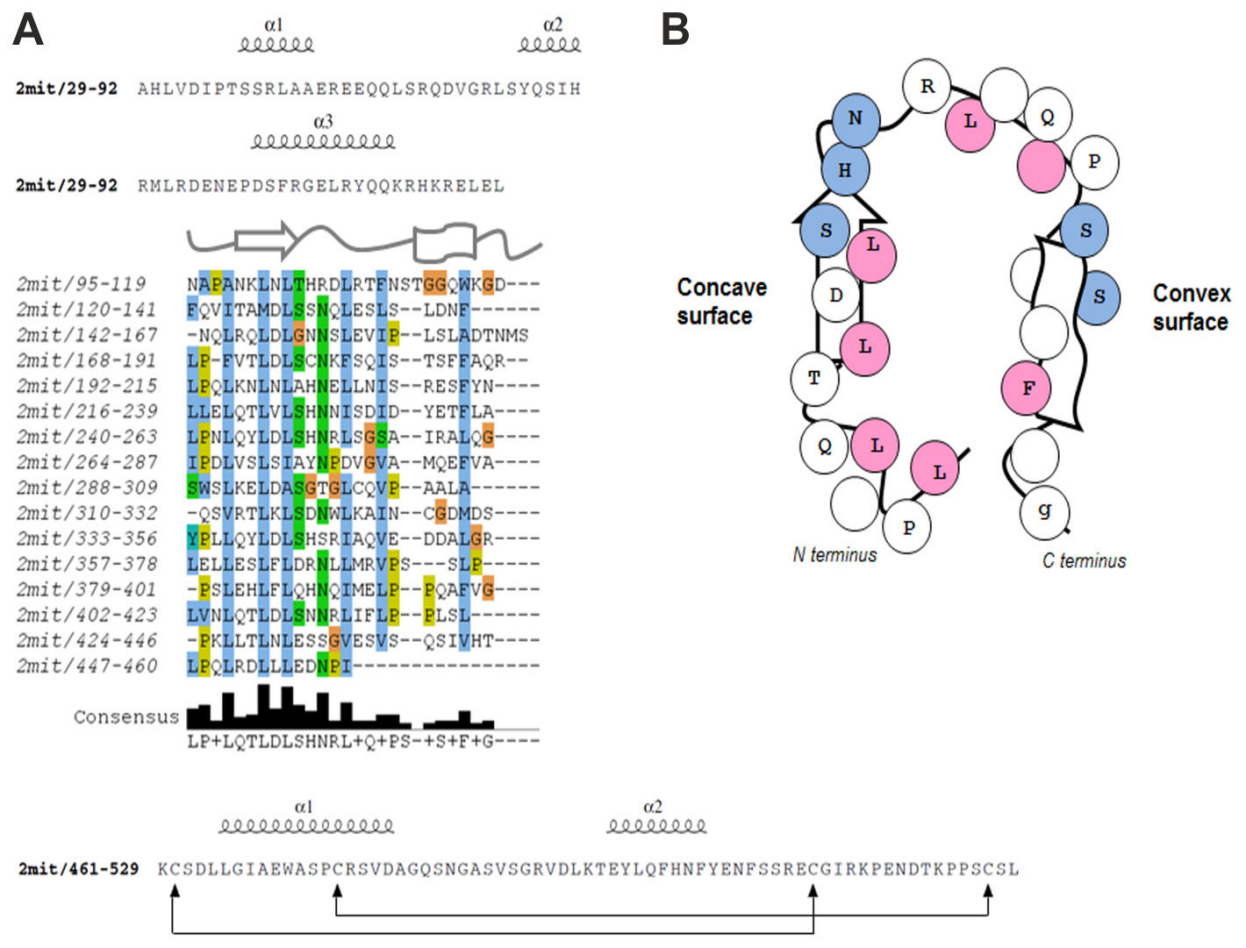
Structural analysis of the *D. melanogaster* 2MIT LRR domain. (A) Identification and structural alignment of the LRR units. The sequence of the LRR N-flanking region forming three hypothetical α-helices is outlined at the top. The main LRR repeats are aligned based on the structural correspondence between residues. A schematic cartoon used to depict the β-strand and the α-helix is shown above, and the consensus sequence is highlighted below the repeats. The bottom shows the LRR C-flanking region sequence with connecting lines between cysteines forming disulfide bonds. (B) Two dimensional representation of LRR repeats with β-strand and α-helix from N- to C-terminus. The consensus sequence is shown inside circles representing single residues. Conserved hydrophobic positions (pink circles) face the internal side of the repeat, while conserved polar residues (blue circles) map on the surface.

A scan of the N-terminal sequence against domain and protein signature databases, such as Pfam and PROSITE, revealed the presence of some LRRs which correspond to structural units (with a LxxLxLxxN/CxL conserved pattern) consisting of a β-strand and an α-helix. Since LRR domains are organized in series, they can form non-globular, crescent-shaped structures, which create a solvent-exposed, elongated, and concave surface of parallel β-strands, acting as a scaffold for interactions with other proteins [10]. The LRR domain was modeled using a MANIFOLD approach [11], which combines the prediction of secondary structures and results obtained by different repeat prediction methods. The first step was to identify the correct number of repeated units. RADAR, TRUST, and Repetita tools revealed different numbers of repeats. A structural alignment of all predicted repeated units was built in order to calculate a consensus pattern and to identify other missing repeats in the region spanning between the 80 and 530 amino acid positions. It was thus possible to recognize 16 repeats matching the consensus xxxLxxLxLxxNxLxxLpxxoFxx sequence that is typical for the LRR domain (Figure 2A). Each repeat contained hydrophobic, conserved positions (mostly Leu residues), predicted to be buried internally and to have a structural role. Other polar/charged residues are likely exposed to the solvent and are probably involved in protein-protein interactions.

Most LRR proteins contain flanking regions that are an integral part of the LRR domain. In the *D. melanogaster* 2MIT protein two terminal variable regions flank the LRR-NT and LRR-CT repeats. These regions usually have a capping role which protects the first and last repeats, but may also have a functional role. The LRR-NT is predicted to be disordered, contains low complexity regions, and includes a cluster of charged residues between the 64 and 91 positions. Secondary structure analysis of the LRR-NT revealed that there are three hydrophilic α-helices which may form a N-terminal cap. The LRR-CT is estimated to be about 70 residues long and contains four Cys that may form disulfide bonds (Figure 2A). The template search identified the structure of the Toll-like receptor 3 (PDB code: 2A0Z, chain A) as the most structurally similar to the 2MIT LRR domain (Figure 2B).

### *2mit* expression during development

**Figure 3:**
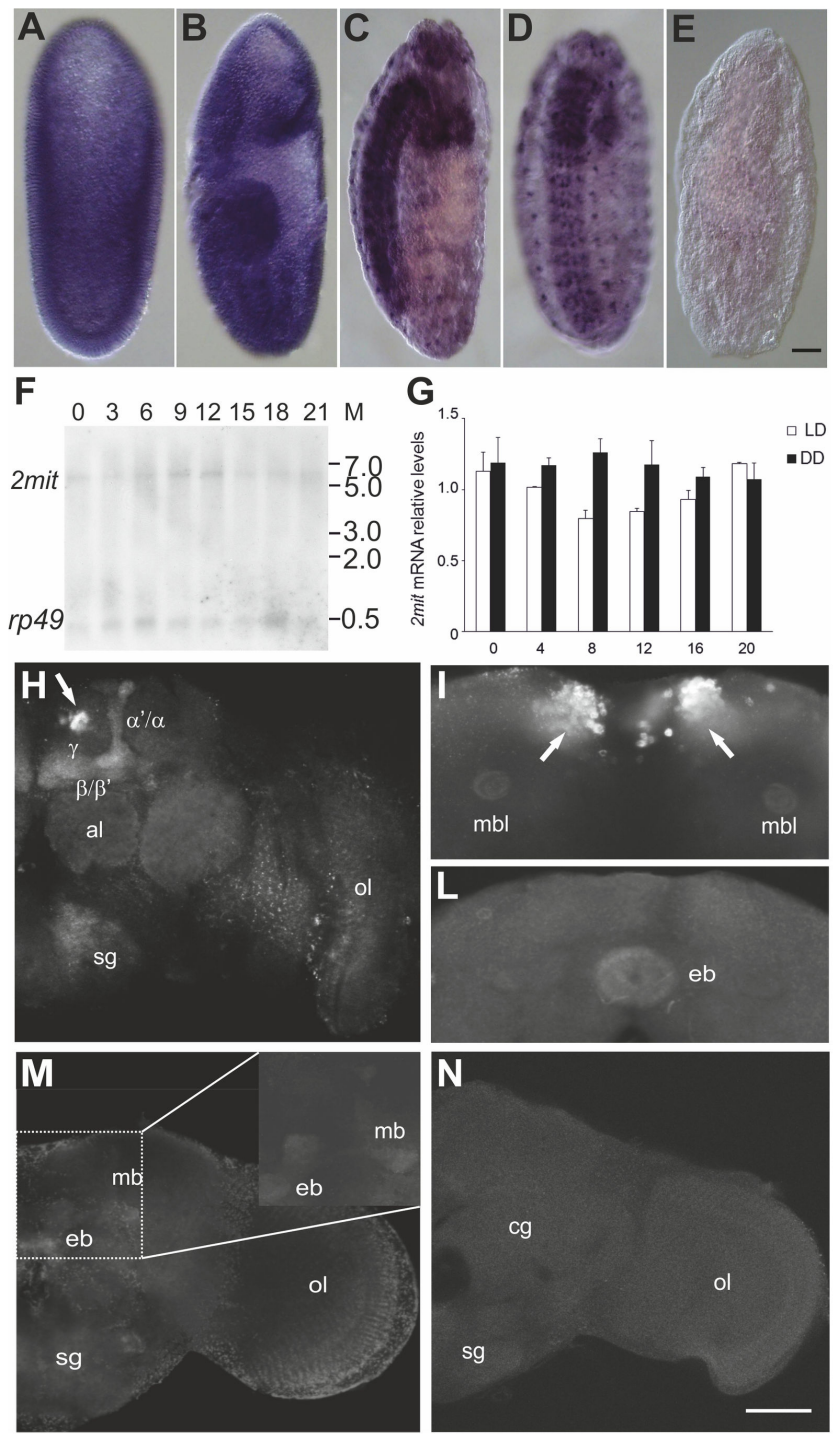
*2mit* mRNA expression in *w* ^1118^ flies. (A-D) Embryos at different developmental stages hybridized with the antisense *2mit* probe. (A) Stage 5. (B) Stage 9. (C-D) lateral and frontal views of stage 15. (E) Negative control showing a stage 15 embryo hybridized with the *2mit* sense probe. Bar in (E) represents 25 µm for (A)-(E). (F) Northern blot from wild-type adult heads sampled every 3 h in 12:12 LD conditions. *2mit* indicates the single *2mit* transcript revealed in adult heads. *rp49* represents the *rp49* housekeeping mRNA. M: RNA Ladder molecular marker. (G) *2mit* mRNA levels [mean ± standard error of the mean (SEM)] sampled every 4 h in 12:12 LD (white bars) and DD (black bars) conditions. For each condition, 3 replicates were performed. In 12:12 LD, significant variations in *2mit* mRNA levels were detected (F_5,10_=7.89, p <0.01). In DD, no significant modifications in *2mit* mRNA levels were detected (F_5,12_= 0.56 p= 0.76). (H-N) *2mit* mRNA localization in whole-mount adult brains sampled at ZT 0. (H-L) Wild-type adult brains hybridized with the *2mit* antisense probe. (H) *2mit* mRNA signals are visible at the level of the Kenyon cells (arrow) and in the different lobes of the MBs (image shows a ~6 µm Z-projection along the antero-posterior axis). (I) and (L) ~5 µm Z-projections obtained from the same brain showing *2mit* mRNA signals in the Kenyon cells (arrows; I) and in the EB (L). (M) *2mit* ^*c03963*^ adult brain hybridized with the *2mit* antisense probe. Weak signals are detected in the MB lobes and EB. (N) Wild-type adult brain hybridized with the *2mit* sense probe (negative control). (M) and (N) are ~10 µm Z-projections. The following abbreviations are used: ol: optic lobe; al: antennal lobe; mb: mushroom bodies; mbl: mushroom bodies lobes; eb: ellipsoid body; sg: subesophageal ganglion; cg: central ganglion. α/ α’: vertical mushroom bodies lobes; β, β’, γ: medial mushroom bodies lobes. Bar in (N) represents 50 µm for (H), (M) and (N), and 25 µm for (I), (L), and the 2X magnification inset in (M).

*2mit* is transcribed throughout *D. melanogaster* development in two mRNA isoforms of ~5.4 and 3.8 kb, carrying a unique 5’ UTR and two length-differing 3’ UTRs (353 and 1892 nt excluding the polyA segment [7]). *In situ* hybridization experiments on whole-mount embryos detected a diffuse *2mit* expression pattern at the blastoderm stage (stage 5; Figure 3A). During segmentation (stage 15), *2mit* mRNA localized in the developing Central and Peripheral Nervous Systems (CNS, PNS; Figure 3C, D).

In adult heads of wild-type flies that underwent 12 h: 12 h light:dark cycles (12:12 LD conditions, with ZTs 0 and 12 corresponding to lights-on and -off, respectively), Northern blot analyses revealed the presence of a single transcript corresponding to the ~5.4 kb *2mit* mRNA isoform, suggesting that at least in that anatomical structure the longer mRNA variant is the most represented *2mit* transcript (Figure 3F). To evaluate potential circadian variations in *2mit* expression levels, quantitative PCR (QPCR) experiments were performed on adult heads, sampled every 4 h during the day, in both 12:12 LD and constant darkness (DD) regimes. Under 12:12 LD conditions, *2mit* transcript levels showed an oscillating profile with a slight but significant variation over the 24 h cycle (F_5,10_= 7.89 p <0.01; Figure 3G). In particular, we revealed an increase in *2mit* mRNA levels at approximately the end of the night/beginning of the day, between ZT 20 and ZT 0, and a reduction at ZT 8 (Newman-Keuls *post-hoc* test: ZT 8 *vs* ZT 0 or ZT 20p<0.05). After two days of DD, *2mit* expression became constitutive (F_5,12_= 0.56 p= 0.76, not significant; Figure 3G).

*2mit* mRNA localization pattern was evaluated by *in situ* hybridization experiments on whole-mount adult fly brains sampled at ZT 0, when *2mit* expression was known to be high. Specific hybridization signals were observed mainly in the MBs and EB of the central complex (Figure 3H-L), structures primarily involved in learning, memory, and locomotor activity control [12,13]. In the MBs, *2mit* mRNA staining was observed in the neuronal somata (Kenyon cells) and at the level of both the vertical (α/α’) and medial (β, β’, γ) lobes, representing compact axonal structures (Figure 3H, I). Additional *2mit* expression was visualized in the sub-esophageal ganglion (SOG; Figure 3H). Diffuse and weak signals were noted in both optic and antennal lobes (OLs, ALs; Figure 3H), probably as a result of non-specific hybridization staining. In fact, similar signals were observed when the *2mit* sense probe was used in the same regions of the negative controls (Figure 3N).

Our *2mit* mRNA analyses are consistent with high-throughput expression data from FlyBase indicating that the highest *2mit* expression levels occur during embryogenesis, between 14 and 20 h after fertilization, and that transcription is restricted to nervous system structures at both larval and adult stages. Moreover, a recent study listed *2mit* (CG 17319) among those genes preferentially expressed in the MBs [14].

### *2mit* is involved in courtship conditioning memory but not in learning

We previously demonstrated that the *c03963* transposon insertion line (Exelixis Gene Disruption Project), carrying a PiggyBac (PB) element ~20 kb upstream of the *2mit* ATG start codon, was characterized by a ~50% *2mit* mRNA decrement and normal *tim2* mRNA levels estimated at the third larval stage (L3 [7];; Figure 1A). After out-crossing with *w* ^1118^ flies for eight generations, a ~50% *2mit* mRNA depletion and unaffected *tim2* mRNA levels compared to *w* ^1118^ controls were confirmed in *c03963* homozygous L3 (*2mit* ^*c03963*^) by QPCR (Table 1). We also analyzed mRNA levels of the other internally transcribed sequences in the *tim2* locus, showing that the *PB* insertion in *2mit* ^*c03963*^ homozygous L3 did not cause any significant modifications in their expression compared to *w* ^1118^controls (mRNA levels in *2mit* ^*c03963*^ and *w* ^1118^ individuals: *CG34308*: 1.13 ± 0.33 *vs* 1 ± 0.0; F_1,6_= 0.15 p= 0.71; *BK002510*: 1.27 ± 0.57 *vs* 1 ± 0.0; F_1,6_= 0.22 p= 0.64; *AY118619*: 0.94 ± 0.17 *vs* 1 ± 0.0; F_1,6_= 0.93 p= 0.37; all p values are not significant). We next determined *2mit* mRNA levels in *2mit* ^*c03963*^ homozygous adult heads, showing ~20% *2mit* mRNA transcription levels compared to those of *w* ^1118^ controls (Table 1). In addition, a weak *2mit* mRNA signal was detected in *2mit* ^*c03963*^ homozygous adult brains by *in situ* hybridization experiments. In particular, we observed faint *2mit* staining in the neuronal fibers of the EB and MB lobes (Figure 3M). No evident *2mit* mRNA signals were detected in the brain region where MB cell bodies are located (Figure 3M), as probably they are under the detection limit of the *in situ* hybridization technique performed in this study.

**Table 1:** Molecular characterization of transposon insertional alleles of the *tim2* locus.

**Genotype**	**Stage**	***2mit* mRNA levels**	***tim2* mRNA levels**
***c03963***	L3	0.54 ± 0.05 (8)^a^	1.26 ± 0.25 (3)
***c03963***	A	0.15 ± 0.03 (9)^b^	nd
***f00075***	L3	0.95 ± 0.13 (2)	0.75 ± 0.14 (2)
***MB03271***	L3	1.28 ± 0.13 (4)	nd
***MB08962***	L3	1.25 ± 0.14 (2)	0.95 ±0.01 (2)
***f06803***	L3	1.30 ± 0.01 (2)	0.25 ± 0.15 (4)^c^
***MB08132***	L3	1.35 ± 0.19 (3)	0.93 ± 0.03 (3)
***w^1118^***	L3	1.29 ± 0.17 (6)	1
***w^1118^***	A	1.28 ± 0.13 (5)	nd

Relative mRNA levels (mean ± SEM) of *2mit* and *tim2* in *c03963, f00075, MB03271, MB08962, f06803*, and *MB08132* homozygous transposon insertional flies compared to *w* ^1118^ controls. (n) indicates numbers of independent replicates. L3: third larval stage; A: adult heads. nd: not determined.

Statistical analyses were performed by comparing *2mit* or *tim2* mRNA levels of homozygous insertional mutant larvae or adult heads with those of *w* ^1118^controls.

a Significantly different *2mit* mRNA levels (F_6,20_ =6.76 p< 0.001). Neuman-Keuls *post* *hoc* test: significantly different *2mit* mRNA levels in *c03963* compared to all other samples at the L3 stage (p< 0.05).

b Significantly different *2mit* mRNA levels in *c03963* adult heads compared to *w* ^1118^controls (F_1,12_ = 130.9 p< 0.0001).

c Significantly different *tim2* mRNA levels (F_5,13_ = 9.31 p<0.001). Neuman-Keuls *post* *hoc* test: significantly different *tim2* mRNA levels in *f06803* L3 compared to all other samples (p< 0.05).

2 *mit* ^c03963^ homozygous flies were viable and fertile. No lethal phenotypes were observed during post-embryonic development (Table 2). Adult 2mit^*c03963*^ homozygous flies did not display any gross morphological abnormalities and their overall brain organization was similar to that of wild-type flies. In addition, they did not show any impairment in light perception, evaluated as phototactic behavior (Table S1), as well as in locomotor activity (Figure S1).

**Table 2:** Effects of *2mit* down-regulation on egg-to-adult viability.

**Parental genotypes**	**E**	**Postembryonic stage genotypes**	**L3**	**L3 Ratio**	**P**	**A**
***2mit*^*c03963*^**	90	*2mitc03963*/*2mit* ^*c03963*^	82	nd	79	76
***2mitKD*^6.1^ X *l(3***)***-31Gal4/*TM6B,*Tb***	132	*l(3*)*-31Gal4>2mit* KD^*6.*1^	49	0.84	40	39
		*2mitKD6*.^1^/TM6B,*Tb*	58		45	41
***2mitKD*^16.2^ X *l(3***)***-31Gal4/*TM6B,*Tb***	349	*l(3*)*-31Gal4>2mit* KD^*16.2*^	153	0.81	137	135
		*2mitKD* ^16.2^ /TM6B,*Tb*	189		156	141
***2mitKD*^61.1^ X *l(3***)***-31Gal4/*TM6B,*Tb***	200	*l(3*)*-31Gal4>2mit* KD^*61.1*^	108	1.2	92	89
		*2mitKD* ^61.1^ /TM6B,*Tb*	90		87	85
***2mitKD*^6.1^ X *elavGal4/*CyO,*Cy***	170	*elavGal4>2mit* KD^*6.1*^	nd	nd	nd	76
		*2mitKD6*.^1^/ CyO,*Cy*	nd		nd	81
***2mitKD*^16.2^ X *elavGal4/*CyO,*Cy***	182	*elavGal4>2mitKD^16.2^*	nd	nd	nd	79
		*2mitKD* ^16.2^ / CyO,*Cy*	nd		nd	82
***2mitKD*^61.1^ X *elavGal4/*CyO,*Cy***	120	*elavGal4>2mit* KD^*61.1*^	nd	nd	nd	45
		*2mitKD* ^61.1^ / CyO,*Cy*	nd		nd	57

Total number of embryos (E), third instar larvae (L3), pupae (P) and adults (A) have been evaluated. For each *2mit* KD X *l(3*)*- 31Gal4/*TM6B,*Tb* genetic cross, at the beginning of third larval stage, the progeny was subdivided based on absence/presence of *Tb* phenotype. Absence of *Tb* phenotype identifies *l(3*)- 31*Gal4*>2*mit* KD individuals; *Tb* marker detects individuals with non-activated *2mit* KD, which represent internal controls of *2mit* KD. +/Tb larval ratio (L3 Ratio) showed that *2mit* down-regulation does not affect embryo-to-larva viability in *l(3*)- 31Gal4>2*mit* KD individuals.

χ^²^ statistical analyses indicated no significant differences between observed and expected numbers for L3, P, and A in progeny derived from each *2mit* KD X *l(3*)*-31Gal4/*TM6B,*Tb* genetic cross (for each comparison: p> 0.05) and for A, in progeny derived from each *2mit* KD X *elavGal4/*CyO,*Cy* genetic cross (for each comparison: p> 0.05).

*2mit* mRNA levels were also analyzed in five other strains (*f00075, f06803, MB03271, MB08962*, and *MB08132*, Exelixis Gene Disruption Project; Figure 1A) carrying *PB* or *Minos* (*MB*) transposons in proximity or within the *2mit* gene. None of these strains showed significant modifications in *2mit* mRNA levels (Table 1); thus they were excluded from subsequent analyses.

**Figure 4:**
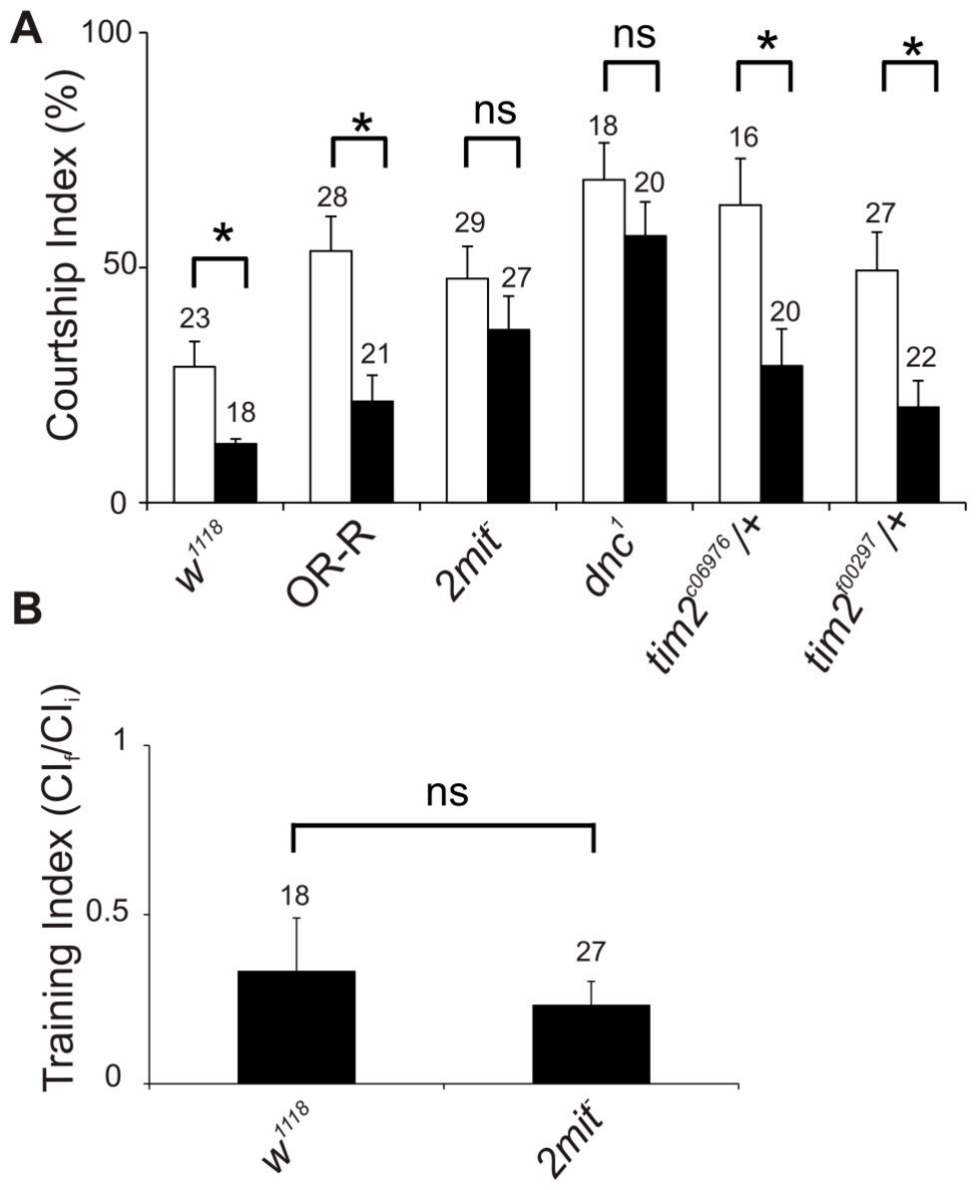
Memory and learning in *2mit* ^*c03963*^ homozygous mutant flies. (A) Comparison between Courtship Indices in sham (white bars) and conditioned (black bars) males for different genotypes. *w* ^1118^ and *OR-R* represent control strains; *2mit* ^*-*^ : *2mit* ^*c03963*^ homozygous mutant flies; *dnc* ^1^: *dunce* ^1^ memory mutant flies; *tim2* ^*c06976*^ */+* and *tim2* ^*f00297*^ */+* heterozygous flies for two different *tim2* ^*-*^ insertional mutant alleles. Data are expressed as mean ± SEM, with the number of tested flies indicated above each bar. Mann-Whitney U test revealed significant differences between the CIs of the conditioned and sham males in *w* ^1118^, *OR-R*, *tim2* ^*c06976*^ */+*, *tim2* ^*f00297*^ */+*: p< 0.05 for all genotypes. No significant differences were identified comparing the CIs of the conditioned and sham males in *2mit* ^*c03963*^ and *dunce* ^1^ flies (*2mit* ^*c03963*^: p=0.38; *dunce* ^1^: p=0.32). (B) Learning evaluated as a training index (ratio between CIs for the last (CI_f_) and the first (CI_i_) 10 min of the training period) in conditioned *2mit* ^*c03963*^ and *w* ^1118^ males (Mann-Whitney U test: p=0.60). *2mit* ^*-*^ : *2mit* ^*c03963*^ homozygous mutant flies. *: significant difference; ns: not significant difference.

Given the specific brain *2mit* mRNA hybridization pattern, we decided to assess whether *2mit* plays a role in the behavioral plasticity associated with learning and memory. Therefore, *2mit* ^*c03963*^ homozygous adult males were analyzed using the courtship conditioning assay [15,16]. This test is based on natural sexual behavior and measures the reduction in courtship levels of male flies which have previously courted non-receptive, mated females [17]. For each genotype, we measured the Courtship Index (CI), defined as the time spent by a male courting an anesthetized virgin female during a 10 min observation period, in conditioned and sham males [15,16]. In *OR-R* control flies, the CIs of conditioned males were significantly reduced compared to those of sham individuals, indicating short-term memory (STM) formation (Mann-Whitney U test: *OR-R*: p<0.01; Figure 4A). As expected, *w* ^1118^ sham males showed lower CI levels compared to *OR-R* sham controls under light conditions since white-eyed, vision-defective males have difficulty tracking females during courtship [18]. However, *w* ^1118^ males showed STM formation, as the CIs of conditioned males were significantly lower with respect to those of sham controls (Mann-Whitney U test: *w* ^1118^: p< 0.05; Figure 4A). Similar results were also described by another study on flies carrying the *w* ^1118^ allele in a cantonized strain (*w CantonS* [19]). Therefore, we considered the *w* ^1118^ flies a suitable control to study memory phenotypes using the courtship conditioning paradigm in *2mit* ^*c03963*^ homozygous flies, which essentially have a *w* ^1118^ genetic background, except for the *PB* transposon insertion detectable by a *mini-w* ^*+*^ marker gene.

The CIs of *2mit* ^*c03963*^ sham males resulted similar to those of *OR-R* sham controls (Mann-Whitney U test: p= 0.36, not significant; Figure 4A), indicating that *2mit* ^*c03963*^ homozygous males possess normal virgin female perception. However, in *2mit* ^*c03963*^ flies, the CIs of conditioned males were not significantly different from those of sham controls (Mann-Whitney U test: p= 0.38, not significant; Figure 4A). Analogous results were obtained after comparing the CIs of sham and conditioned males in the classic memory mutant *dunce* (*dnc* ^*1*^), used as negative control (Mann-Whitney U test: p= 0.32, not significant; Figure 4A). Since during the test we did not observe evident impairments in courtship behavior and any abnormality in locomotor activity of *2mit* ^*c03963*^ flies (Figure S1), these data suggest the presence of memory defects in *2mit* ^*c03963*^ mutant males.

In addition, when *tim2* ^*-*^/+ heterozygous males for two different alleles (*tim2* ^*c06976*^ and *tim2* ^*f00297*^) have been analyzed, the CIs of conditioned males resulted significantly lower compared to those of sham controls (Mann-Whitney U test: p< 0.05 for both *tim2* ^*c06976*^/+ and *tim2* ^*f00297*^/+ individuals; Figure 4A), suggesting that the *tim2* depletion in *tim2* ^*-*^/+ heterozygous flies does not cause memory impairment.

To understand whether *2mit* ^*c03963*^ memory defects were associated with training (learning) deficiencies, we determined the training index (TI), defined as the ratio between CIs during the final (CI_f_) and initial 10 min (CI_i_) of the training period [15,16], in conditioned *2mit* ^*c03963*^ mutant and *w* ^1118^ control males. TI values ≤ 0.5 are generally typical of wild-type flies, while TIs > 0.5 are characteristic of conditioning defective individuals [16]. Both *2mit* ^*c03963*^ and *w* ^1118^ flies exhibited TIs < 0.5 and the comparison between *2mit* ^*c03963*^ and *w* ^1118^ did not show any significant difference (Mann-Whitney U test: p= 0.6, not significant; Figure 4B), indicating that *2mit* ^*c03963*^ males reduced their courtship behavior in the presence of a mated female, thus displaying learning activity.

**Figure 5:**
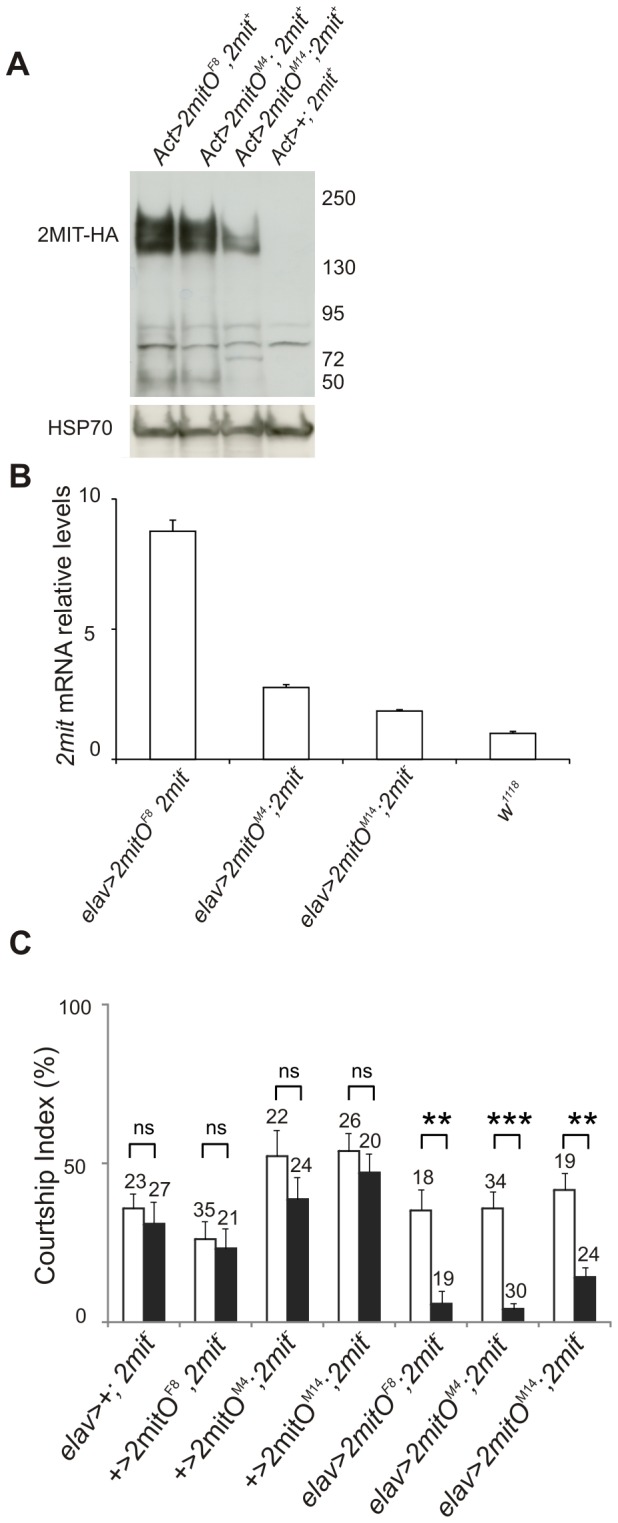
*2mit* over-expression in adult flies. (A) 2MIT-HA Western blot on adult bodies of *ActGal4>2mitO* ^*F8*^, *ActGal4>2mitO* ^*M4*^, *ActGal4>2mitO* ^*M14*^ and negative control *ActGal4*>+, in a wild-type *2mit* ^*+*^ background. 2MIT-HA indicates the ~150 KDa 2MIT-HA form revealed in all *ActGal4>2mitO* over-expressing lines. HSP70 represents ~70 KDa Heat Shock Protein 70, used as loading control. Four replicates were performed. (B) *2mit* mRNA relative levels (mean ± SEM) in dissected brains of three independent *elavGal4>2mitO; 2mit* ^*c03963*^ lines (*F8, M4* and *M14*) and in *w* ^1118^ controls. Plot of 9 replicates. F_3,31_= 552 p< 0.0001; Newman-Keuls *post-hoc* test: each *elavGal4>2mitO; 2mit* ^*c03963*^ line *vs* *w* ^1118^ controls p< 0.001. (C) Courtship Indices in sham (white bars) and conditioned (black bars) males for the *elavGal4>2mitO, 2mit* ^*c03963*^ lines (*F8, M4* and *M14*) and relative negative controls *elavGal4>+; 2mit* ^*c03963*^; and + > *2mitO ; 2mit* ^*c03963*^ (*F8, M4* and *M14*). *2mit* ^*-*^ indicates the *2mit* ^*c03963*^ allele. Data are expressed as mean ± SEM, with the number of tested flies indicated above each bar. The CIs of conditioned flies were not significantly different from those of sham males in the negative control lines (Mann-Whitney U test: *elavGal4>+; 2mit* ^*c03963*^: p= 0.24 ; + >*2mitO* ^*F8*^ *, 2mit* ^*c03963*^: p= 0.81; + >*2mitO* ^*M4*^ *; 2mit* ^*c03963*^: p= 0.33; + >*2mitO* ^*M14*^ *; 2mit* ^*c03963*^: p= 0.43), but were significantly reduced in *elavGal4>2mitO, 2mit* ^*c03963*^ lines. The number of asterisks indicates the significance level: **: p < 0.005; ***: p < 0.0001; ns: not significant.

In order to determine whether memory defects in *2mit* ^*c03963*^ flies were related to *2mit* depletion, we generated three independent transgenic lines (*2mitO* ^*F8*^ *, 2mitO* ^*M4*^, *2mitO* ^*M14*^) for a UAS-*2mitHA* cDNA chimeric construct designed for *2mit* over-expression studies. The presence of a 2MIT-HA chimeric protein was visualized by Western blot from flies in which 2MIT-HA over-expression was ubiquitously activated using an *Actin5C-Gal4* (*ActGal4*) driver in a wild-type *2mit* ^*+*^ genetic background (Figure 5A). We identified a ~150 kDa band in the three *ActGal4*> *2mitO* lines that was absent in the negative control (*ActGal4>CyO*) and likely represents 2MIT-HA. The discrepancy in molecular weight between the visualized band and the one expected from the estimation of 2MIT-HA theoretic weight (~122 kDa) may have resulted from anomalous detergent binding and denaturation in SDS-PAGE migration, which for transmembrane proteins may explain till ± ~45% molecular weights variations [20]. The three *ActGal4>2mitO* lines overexpressed different degrees of the 2MIT-HA protein, with higher levels in *ActGal4>2mitO* ^*F8*^flies and lower ones in *ActGal4>2mitO* ^*M14*^ individuals.

Both *2mit* mRNA and 2MIT-HA chimeric protein productions were then evaluated in adult brains of flies over-expressing the UAS-*2mitHA* construct at the level of the MBs (using the OK107*Gal4* driver) in a wild-type *2mit* ^*+*^ genetic background. In OK107*Gal4*>*2mitO* brains, *2mit* mRNA and 2MIT-HA protein signals co-localized in the Kenyon cells and axonal lobes of the MBs (Figure S2).

Using genetic crossing and the pan-neuronal *elavGal4* driver, we generated three *elavGal4*>*2mitO* lines, over-expressing 2MIT at the CNS level in a mutant 2mit^*c03963*^ background (*elavGal4>2mitO* ^*F8*^, *2mit* ^*c03963*^ *; elavGal4>2mitO* ^*M4*^ ; *2mit* ^*c03963*^ and *elavGal4>2mitO* ^*M14*^; *2mit* ^*c03963*^). *2mit* mRNA levels were then checked in dissected adult brains by QPCR, which revealed *2mit* mRNA over-expression in all three *elavGal4>2mitO*; *2mit* ^*c03963*^ transgenic lines compared to *w* ^1118^ controls, with higher values in *elavGal4>2mitO* ^*F8*^, *2mit* ^*c03963*^ flies (~ 9-fold higher) and lower in *elavGal4>2mitO* ^*M14*^ ; *2mit* ^*c03963*^ individuals (~ 2-fold higher; Figure 5B).

In order to evaluate 2MIT’s ability to rescue the *2mit* ^*c03963*^mutant memory phenotype, we determined the CIs of conditioned and sham male flies for the three *elavGal4*>*2mitO, 2mit* ^*c03963*^ lines (*elavGal4>2mitO* ^*F8*^ *, 2mit* ^*c03963*^ *; elavGal4>2mitO* ^*M4*^ *; 2mit* ^*c03963*^ and *elavGal4>2mitO* ^*M14*^ *; 2mit* ^*c03963*^) and relative controls (*elavGal4>+; 2mit* ^*c03963*^; +>*2mitO* ^*F8*^ *, 2mit* ^*c03963*^; +>*2mitO* ^*M4*^ *; 2mit* ^*c03963*^; +>*2mitO* ^*M14*^ *; 2mit* ^*c03963*^; Figure 5C). In all three *elavGal4*>*2mitO, 2mit* ^*c03963*^ lines, the CIs of conditioned males were significantly reduced with respect to those of sham individuals, indicating a substantial STM rescue (Mann-Whitney U test: *elavGal4>2mitO* ^*F8*^, *2mit* ^*c03963*^: p< 0.005; *elavGal4>2mitO* ^*M4*^; *2mit* ^*c03963*^: p< 0.0001; *elavGal4>2mitO* ^*M14*^; *2mit* ^*c03963*^: p< 0.005). Memory defects were maintained in negative control flies carrying the *elavGal4* driver or the *UAS-2mitHA* construct alone in a mutant 2mit^*c03963*^ background, with the CIs of conditioned males similar to those of sham flies in all control lines (Mann-Whitney U test: *elavGal4>+; 2mit* ^*c03963*^: p= 0.24; + >*2mitO* ^*F8*^ *, 2mit* ^*c03963*^: p= 0.81; + >*2mitO* ^*M4*^ *; 2mit* ^*c03963*^: p= 0.33; + >*2mitO* ^*M14*^ *; 2mit* ^*c03963*^: p= 0.43; all p values are not significant). Taken together, these data suggest that *2mit* is required for *Drosophila* memory formation.

### *2mit* downregulation alters memory phenotype

**Figure 6:**
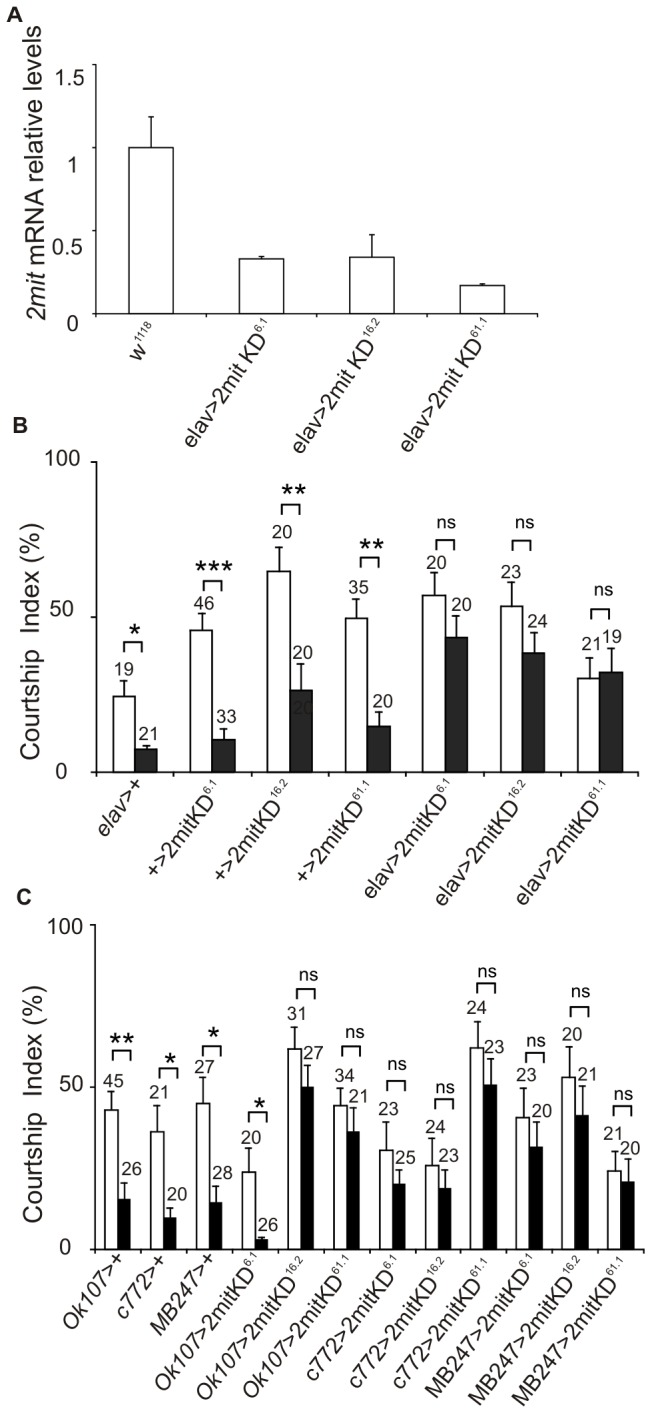
*2mit* knockdown in adult flies. (A) *2mit* mRNA relative levels (mean ± SEM) in the dissected brains of three independent *elavGal4>2mit*KD (*6.1*; *16.2*; *61.1*) and *w* ^1118^ controls. Plot of 6 replicates. F_3,20_= 1571, p<0.0001; Newman-Keuls *post-hoc* test: each *elavGal4>KD* line *vs* *w* ^1118^ controls: p< 0.001. (B) Courtship Indices in sham (white bars) and conditioned (black bars) males for *elavGal4>2mit*KD (*6.1*; *16.2*; *61.1*) and appropriate controls [*elavGal4>+*; +> *2mit* KD (*6.1*; *16.2*; *61.1*)]. Mann-Whitney U test showed no significant differences between the CIs of sham and conditioned males in all the *elavGal4>2mit* KD (*elavGal4*>*2mit* KD^*6.1*^.: p= 0.17; *elavGal4*>*2mit* KD^*16.2*^: p= 0.13; *elavGal4*>*2mit* KD^*61.1*^: p= 0.88). Significant differences between the CIs of conditioned and sham males were found in the control lines *elavGal4*> +; +> *2mit* KD^*61*^. ; +> *2mit* KD^*16.2*^ ; +> *2mit* KD^*61.1*^ ; +> *2mit* KD^*61.1*^. (C) Courtship Indices in sham (white bars) and conditioned (black bars) males for OK107*Gal4>2mit*KD (*6.1*; *16.2*; *61.1*), c772*Gal4>2mit*KD (*6.1*; *16.2*; *61.1*), MB247*Gal4>2mit*KD (*6.1*; *16.2*; *61.1*) lines, and the control lines OK107*Gal4>+*, c772*Gal4>+*, MB247*Gal4>+*. Mann-Whitney U test showed no significant differences between the CIs of sham and conditioned males in two out of three OK107*Gal4>2mit* KD lines (OK107*Gal4>2mit* KD^*16.2*^: p= 0.18; OK107*Gal4>2mit* KD^*61.1*^: p= 0.36), in all the c772*Gal4>2mit* KD flies (c772*Gal4*>*2mit* KD^*61*^. : p= 0.67; c772*Gal4*>*2mit* KD^*16.2*^: p= 0.72; c772*Gal4*>*2mit* KD^*61.1*^: p= 0.42) and in all the three MB247*Gal4>2mit* KD lines (M247*Gal4*>*2mit* KD^*61*^. : p= 0.88; MB247*Gal4*>*2mit* KD^*16.2*^: p= 0.12; MB247*Gal4*>*2mit* KD^*61.1*^: p= 0.27). Significant differences between the CIs of conditioned and sham males were found in the control lines OK107*Gal4>+*, c772*Gal4>+*, MB247*Gal4>+* and in the OK107*Gal4>2mit* KD^*6*1^. transgenic line. In (B) and (C) data are expressed as mean ± SEM, with the number of tested flies indicated above each bar. The number of asterisks indicates the significance level: *: p < 0.05; **: p < 0.005; ***: p < 0.0001; ns: not significant.

To confirm the involvement of *2mit* in STM formation, we produced three independent transgenic lines (*2mit* KD^*6.1*^; *2mit* KD^*16.2*^; *2mit* KD^*61.1*^) carrying a UAS-construct for *2mit* knock-down (KD) analyses. No lethal phenotypes were observed when *2mit* KD was generally induced in both neuroblasts and neurons (with the *l(3*)*-31Gal4* driver) or in mature neurons (with the *elavGal4* driver) during embryonic development (Table 2). Pan-neuronal *elavGal4>2mit* KD produced different levels of *2mit* mRNA depletion in dissected adult brains of the three transgenic lines. In fact, *2mit* expression levels ranged from 0.17 ± 0.01 in *2mit* KD^*61.1*^ to 0.34 ± 0.01 in *2mit* KD^*6*.1^ compared to 1.00 ± 0.19 of *w* ^1118^ controls (Figure 6A).

The STM phenotype in the *elavGal4>2mit* KD lines was then analyzed (Figure 6B). The pan-neuronal *2mit* mRNA KD affected STM formation, since the CI values of conditioned flies resulted comparable to those of sham males in all of the *elavGal4>2mit* KD lines (Mann-Whitney U test: *elavGal4*>*2mit* KD^*6.1*^ **: p= 0.17; *elavGal4*>*2mit* KD^*16.2*^: p= 0.13; *elavGal4*>*2mit* KD^*61.1*^: p= 0.88; all p values are not significant). Moreover, significant differences were observed in the CIs of conditioned and sham flies in all negative controls (Mann-Whitney U test: *elavGal4*> +: p< 0.05; +> *2mit* KD^*6*.1^ : p< 0.0001; +> *2mit* KD^*16.2*^ and +> *2mit* KD^*61.1*^ : p< 0.005), suggesting once again that *2mit* plays a specific role in the *Drosophila* memory phenotype.

We then evaluated the CIs of conditioned and sham males in transgenic flies, in which *2mit* was silenced mainly in the whole MB structure, using the OK107- and c772*Gal4* drivers, or in α, β, and γ MB lobes, with the MB247*Gal4* driver [21]. Additional *2mit* KD could be produced in other brain regions, since these drivers result weakly active also in the OLs and ALs (OK107*Gal4*), the OLs, ALs, EB and SOG (c772*Gal4*), and the OLs and glia cells (MB247*Gal4*) [21].

In two out of three OK107*Gal4>2mit* KD transgenic lines (*2mit* KD^*16.2*^ and *2mit* KD^*61.1*^) and in all of the c772*Gal4>2mit* KD and MB247*Gal4> 2mit* KD lines (*2mit* KD^*6.1*^ *, 2mit* KD^*16.2*^, and *2mit* KD^*61.1*^), the CIs of conditioned and sham males were not statistically different, indicating altered memory formation (Mann-Whitney U test: with the OK107*Gal4* driver*: 2mit* KD^*16.2*^: p= 0.18; *2mit* KD^*61.1*^: p= 0.36; with the c772*Gal4* driver*: 2mit* KD^*6.1*^ **: p= 0.67; *2mit* KD^*16.2*^: p= 0.72; *2mit* KD^*61.1*^: p= 0.42; with the MB247*Gal4* driver*: 2mit* KD^*6.1*^ **: p= 0.88; *2mit* KD^*16.2*^: p= 0.12; *2mit* KD^*61.1*^: p= 0.27; all p values are not significant; Figure 6C). On the contrary, normal STM was observed in all the appropriate negative controls (+> *2mit* KD lines; Figure 6B; Mann-Whitney U test: OK107*Gal4*> +: p<0.005; c772*Gal4*> + and MB247*Gal4*> +: p< 0.05; Figure 6C). The third OK107*Gal4>2mit* KD^*6.*1^ transgenic line did not show any impairment in the STM phenotype, since in that case the CIs of conditioned males were significantly reduced from those of sham controls (Mann-Whitney U test: p< 0.05; Figure 6C). The absence of STM defects in that line could have been due to an inefficient *2mit* downregulation generated by the combination of the OK107*Gal4* driver and the *2mit* KD transgene, specifically in OK107*Gal4> 2mit* KD^*6*.1^ flies. In fact, when *2mit* KD was pan-neuronally activated in *2mit* KD^*6.*1^ flies by *elavGal4*, a *2mit* mRNA down-regulation in dissected brains and parallel defects in STM were detected. In addition, the use of both c772- and MB247*Gal4* drivers produced STM deficiencies in *2mit* KD^*6.*1^ flies.

Finally, no STM deficiencies were detected when *2mit* silencing was induced in different neurons of the central complex, using the c232- and 52Y*Gal4* drivers. In fact, the CIs of conditioned males were significantly reduced compared to those of sham controls for all these *2mit* KD-driver combinations (Mann-Whitney U test: c232*Gal4*> + and c232*Gal4> 2mit* KD^*6.1*^: p< 0.005; c232*Gal4*> *2mit* KD^*16.2*^: p< 0.0001; c232*Gal4*> *2mit* KD^*61.1*^: p< 0.05; 52Y*Gal4*>+ and 52Y*Gal4> 2mit* KD^*6.1*^ **: p<0.005; 52Y*Gal4*> *2mit* KD^*16.2*^: p< 0.05; 52Y*Gal4*> *2mit* KD^*61.1*^: p< 0.0001; Figure S3). These data rule out the R3 and R4d neurons (active in the c232*Gal4* driver [22]), as well as the R1, R3 and R4 neuronal cells and the subtype of F neurons connecting the fan-shaped body to the EB (active in the 52Y*Gal4* line [22]) as possibly involved in the *2mit*–mediated STM phenotype.

### The nested *2mit* gene does not show a functional relationship with its *timeless2* host gene

**Figure 7:**
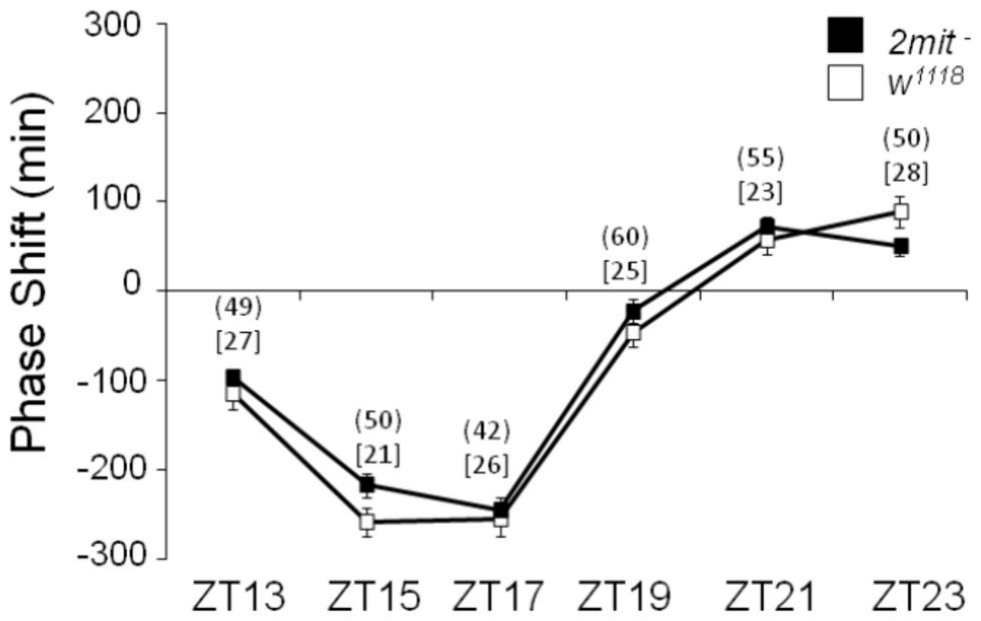
Phase response curve of *2mit* ^*c03963*^ and *w* ^1118^ flies. Analysis of variance genotype X time interactions (ZT): F_5,434_=1.76 p=0.12, not significant. Advance and delay phase shift responses are represented respectively as positive and negative values. *2mit* ^*-*^: *2mit* ^*c03963*^ homozygous mutant flies. Data are expressed as mean ± SEM; (Ns) and [Ns] indicate the number of *2mit* ^*c03963*^ and *w* ^1118^ flies analyzed, respectively.

A functional relationship could be shared by nested and host genes [5]. In *Drosophila* , homozygous *tim2* ^*-*^ */tim2* ^*-*^ flies die very early during pupal development. As heterozygous *tim2* ^*-*^ */+* adult flies are characterized by a modified light synchronization response of the circadian clock [7], we investigated whether *2mit* shares similar functions in the adult brain. Since the natural *ls/s* polymorphism in the circadian clock gene *tim1* significantly influences circadian light responses [23,24], we initially determined the *ls-tim1/s-tim1* genotype, showing that both *2mit* ^*c03963*^ and *w* ^1118^ flies were homozygous for the *ls-tim1* variant. Subsequently, we analyzed *2mit* ^*c03963*^ and *w* ^1118^ adult male responses to 20 min light pulses given at different times during the night (ZTs: 13, 15, 17, 19, 21, and 23), generating a phase response curve (PRC). As expected, *w* ^1118^ control flies showed delayed or advanced phase shifts in locomotor activity when pulsed at the beginning (ZTs: 13, 15, and 17) or at the end (ZTs: 21 and 23) of the night, respectively (Figure 7). The PRC profile of *2mit* ^*c03963*^ flies was similar to that of *w* ^1118^ controls (genotype X time (ZT) interactions: F_5,434_=1.76, p=0.12, not significant; Figure 7), suggesting that *2mit* is not involved in light synchronization of the adult fly circadian clock.

### *Drosophila melanogaster* 2MIT orthologs and phylogenesis

Searching for 2MIT orthologs with tBlastN (using *D. mel* 2MIT as a query) against non-redundant sequences identified 2MIT conservation in 12 *Drosophilidae* genomic sequences released in 2007 (*D. melanogaster,* *D* *. simulans* *,* *D. sechellia* *,* *D. yakuba* *,* *D* *. erecta* *,* *D. ananassae* *,* *D* *. pseudoobscura* *,* *D* *. persimilis* *,* *D. willistoni* *,* *D* *. mojavensis* *, D. virilis*, and *D* *. grimshawi* ; *Drosophila* 12 Genomes Consortium 2007, http://rana.lbl.gov/drosophila/), in 4 *Lepidoptera* (*Manduca sexta* and *Bombyx mori* moths and *Danaus* *plexippus* and *Heliconiusmelpomene* butterflies) and in the following insect species: *Culex* *quinquefasciatus* , *Apis mellifera*, *Aedes aegypti*, *Pediculus* *humanus* , and *Acyrthosiphon pisum*. Due to the presence of gaps in the assembly of contigs, the multi-alignment process of 2MIT orthologs showed an incomplete coverage in 8 *Drosophilidae* species ( *D* *. simulans* *,* *D. sechellia* *,* *D. yakuba* *,* *D* *. erecta* *,* *D. ananassae* *,* *D* *. pseudoobscura* *,* *D* *. persimilis* , and *D* *. willistoni* ) and in all the non-*Drosophilidae* species, except for *A. mellifera*. It was possible to extend the *2mit* coding region for *Drosophilidae* and *Lepidoptera*, using the Augustus gene prediction tool [25] on available whole-genome-shotgun sequences, and a full-length *2mit* coding sequence was obtained (Table 3). To corroborate gene prediction results, each identified full-length *2mit* coding region was then subjected to a Blast *vs* dbEST search in order to verify at least partial coverage by specific expressed sequence tags (ESTs). We subsequently extended analyses to 9 new available *Drosophilidae* sequenced genomes released in 2013 [including only whole-genome-sequences (wgs) data; https://www.hgsc.bcm.edu/content/drosophila-modencode-project] and ran the Augustus gene prediction tool for each contig identified by tBlastN. 2MIT orthologs were detected in *D. eugracilis* *,* *D. rhopaloa* *,* *D. biarmipes* *,* *D. bipectinata* *,* *D* *. elegans* *,* *D. ficusphila* *,* *D. kikkawai* *,* *D* *. takahashii* , and *D* *. miranda* (Table 3). However, it was not possible to obtain a full-length *2mit* coding region for *C* *. quinquefasciatus* , *A. aegypti*, *P* *. humanus* and *A* *. pisum* , since no additional sequence annotations were available on the National Center for Biotechnology Information (NCBI) database; thus they were excluded from subsequent analyses.

**Table 3:** Protein information on 2MIT identified orthologs.

**Gene ID**	**Species**	**Protein**	**Predicted protein**	**I %**	**S %**
*FBgn0260793	*D. melanogaster*	1141	1141	100	100
*FBgn0126657	* D. grimshawi *	1177	1177	62	77
*FBgn0080630	* D. pseudoobscura *	822	1158	66	74
*FBgn0198065	*D. virilis*	1160	1160	62	74
*FBgn0146946	* D. mojavensis *	1117	1117	56	65
*FBgn0241583	* D. yakuba *	824	1153	95	98
*FBgn0221118	* D. willistoni *	823	1162	66	74
*FBgn0109306	* D. erecta *	811	1140	87	90
*FBgn0191991	* D. simulans *	798	1001	93	94
*FBgn0096918	* D. ananassae *	812	1138	71	78
*FBgn0160729	* D. persimilis *	817	1161	75	63
*FBgn0180817	* D. sechellia *	411	1084	71	75
§AFPQ01007265	* D. eugracilis *	NA	1157	85	89
§AFPP01023804	* D. rhopaloa *	NA	1147	91	96
§AFFD01004651	* D. biarmipes *	NA	999	90	94
§AFFE01008324	* D. bipectinata *	NA	1141	74	80
§AFFF01004256	* D. elegans *	NA	1154	83	88
§GL987664	* D. ficusphila *	NA	1188	81	85
§JH111146	* D. kikkawai *	NA	1144	77	82
§JH112787	* D. takahashii *	NA	1143	80	85
§AJMI02003167	* D. miranda *	NA	1155	65	73
£GB10366	*A. mellifera*	999	999	36	53
£HMEL002549	* H. melpomene *	NA	825	36	53
£nscaf3048	*B. mori*	NA	802	39	57
£JH387114	* D. plexippus *	NA	710	39	53
§AIXA01000612	*M. sexta*	NA	792	38	57

Annotated and predicted protein products are listed (numbers indicate aa length). I %: % Identity; S %: % Similarity; *: FlyBase ID; £: Ensembl; § GenBank ID; NA: not available.

We generated a 2MIT ortholog multi-alignment for the 21 *Drosophilidae*, 4 *Lepidoptera* and *Apis mellifera* species, detecting a significant degree of conservation (Table 3 and File S1). In all 2MIT proteins, the N-terminal signal peptide, LRR domains, and transmembrane helix were identified (File S1). In *Drosophilidae*, both the disordered central region and C-terminal domain were longer compared to those of other 2MIT orthologs. Moreover, the 2MIT putative LRR domains of *Lepidoptera* and *A. mellifera* were characterized by the presence of two additional repeated units.

**Figure 8:**
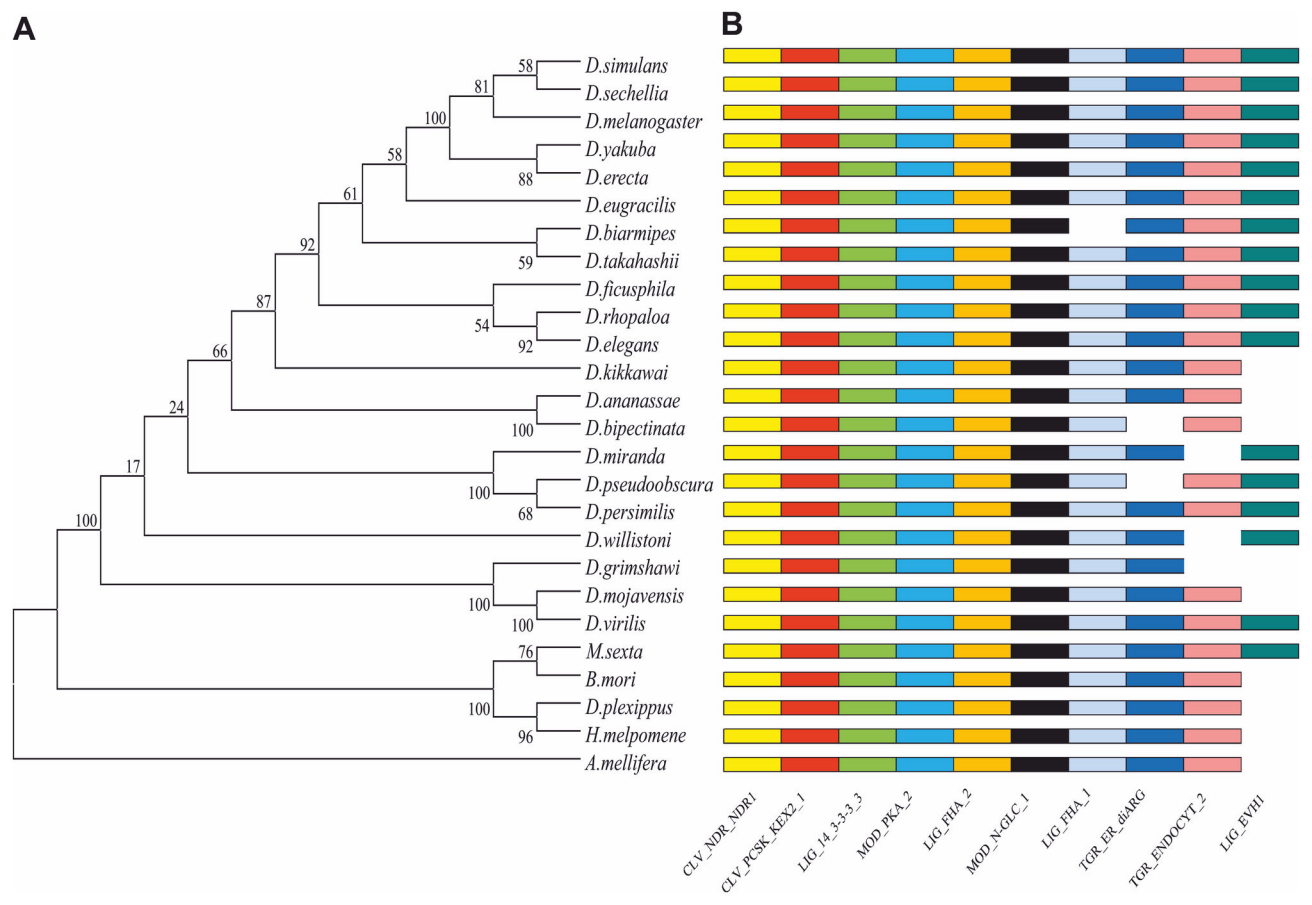
2MIT Phylogenetic analysis. (A) Unrooted phylogenetic tree of 2MIT protein in 21 *Drosophilidae*, 4 *Lepidoptera* and *Apis mellifera*. Statistical support for nodes on the trees was evaluated by the bootstrapping values (×500) shown in each branch point obtained by the Maximum-Likelihood method included in the MEGA 5.0 Software. (B) Colored bars at the right side of the phylogenetic tree represent binding motifs recognized by different factors in 2MIT ortholog cytoplasmic regions. CLV_NDR_NDR1: N-arginine dibasic convertase site; CLV_PCSK_KEX2_1: subtilisin-like proprotein convertases cleavage site; LIG_14-3-3_3: 14-3-3 ligand site; MOD_PKA_2: PKA Phosphorylation site; LIG_FHA_1 and LIG_FHA_2: FHA phosphopeptide ligands; MOD_N-GLC_1: N-glycosylation site; TRG_ER_diArg_1: di Arginine retention/retrieving signal; TRG_ENDOCYTIC_2: sorting signal motif; LIG_EVH1_1: EVH1 ligands.

Maximum Likelihood analysis produced a 2MIT phylogenetic tree which mirrored the species tree. Among *Drosophilidae*, 2MIT phylogenesis followed both subgenus and subgroup classification of the *Drosophila* genus (Figure 8A).

Within the 2MIT cytoplasmic region, the ELM server [26] identified different binding motifs common to almost all species (Figure 8B). Descriptions for these linear motifs can be found on the ELM web-site. In particular, in all 2MIT orthologs we detected recognition sites for N-arginine dibasic convertase (CLV_NDR_NDR1) and subtilisin-like pro-protein convertases (CLV_PCSK_KEX2_1), known to be involved in post-transduction maturation of several target proteins, as well as ligand sites for 14-3-3 protein/s (LIG_14-3-3_3) and a PKA phosphorylation site (MOD_PKA_2), implicated in fundamental cellular processes such as signal transduction and cell-cycle control. Moreover, at least one ligand site for FHA factors (LIG_FHA_1 and/or LIG_FHA_2), a protein domain involved in many signaling processes, and one intracellular sorting signal motif (TRG_ENDOCYTIC_2 and/or TRG_ER_diArg_1) were found in all the 2MIT ortholog C-terminal regions analyzed.

### Analyses of *tim2-2mit* chromosomal organization in *Drosophilidae*, *Lepidoptera* and *Apis mellifera*

Comparative analyses of the *D. melanogaster 2mit*-harboring chromosomal region with those of the 12 *Drosophila* species sequenced in 2007 made it possible to identify conserved synteny using a 200kb-gene sliding window. Specifically, in all 12 species, *2mit* was found to localize in an opposite transcriptional orientation within the *tim2* 11^th^ intron. Moreover, *2mit* was always identified in an opposite transcriptional direction in the same contig of the *tim2* gene in the 9 newly sequenced *Drosophila* species released in 2013, which presented only wgs data. Analyses of the *A. mellifera* genome database (release 4.5) showed that the *2mit* honey bee (*Am 2mit*) ortholog maps internally to the *Am tim2* intron 14 on opposite strands. However, the *tim2*-*2mit* host-nested gene structure was not maintained in the 4 lepidopterous species (EnsemblMetazoa, release 19).

Finally, we analyzed the *D. melanogaster* genomic region around the *c03963* transposon, using a sliding window of approximately 400 nucleotides both upstream and downstream of the insertion site, searching for potential transcription factors (TFs). In particular, we identified a 6 bp TF binding site specific for the zinc-finger neuronal SNAIL repressor within the 11^th^ intron of the *D. melanogaster tim2* gene (5'CAGGTG3', mapping ~20 kb upstream of the *2mit* coding region). Comparative analyses performed on the 21 *Drosophilidae* identified SNAIL binding site conservation in the *tim2* 11^th^ intron in all species with the exception of *D. virilis,* *D. ananassae* *,* *D* *. grimshawi* , and *D* *. rhopaloa* , which are characterized by incomplete assembly coverage in the region upstream of *2mit*.

## Discussion

*2mit* was originally identified as an intronic protein-coding gene located in an opposite transcriptional orientation within the 11^th^ intron of the *Drosophila melanogaster tim2* locus. Its transcription at all developmental stages gives rise to two *2mit* mRNA variants with different 3’ UTR lengths (~5.4 and ~3.8 kb [7]). A recent FlyBase release (FB2013_03) suggested that *AY118619* is part of the *2mit* gene, representing a portion of the 1.3 kb *2mit* 5’ UTR. This conceptually inferred analysis predicts that *2mit* is composed of 4 exons and 3 introns and transcribed in a ~8.8 kb mRNA, ~3 kb longer than the unique ~5.4 kb *2mit* mRNA isoform detected by Northern blot in adult fly heads. Furthermore, *2mit* ^*c03963*^ homozygous flies carrying the *PB* transposon ~20 kb upstream of the *2mit* coding region and in the second intron of the *AY118619* sequence have shown marked *2mit* mRNA depletion and unaltered *AY118619* mRNA levels. These data suggest that *2mit* and *AY118619* are two independent embedded genes in the *tim2* locus and confirm our previous results indicating that *2mit* is organized in 2 exons and 1 intron [7].

*In silico* analysis has indicated that *D. melanogaster 2mit* encodes a transmembrane protein carrying a LRR domain of 16 repeats in the extracellular portion and a Ser-rich region in the cytoplasmic portion, which might represent a putative binding site for different molecular factors. LRR domains are widespread and highly conserved structural motifs with a primary function in protein-protein interactions [10]. LRR-bearing proteins in Eukaryotes have been shown to be key components in several biological processes, such as embryonic development, cell adhesion and signaling, and extracellular matrix assembly [27]. Among Metazoans, LRR proteins are fundamental in neuronal circuit development, including axon/dendrite guidance and synapse formation [28]. In particular, both transmembrane and secreted LRR proteins seem to play a key role in the alignment of pre- and post-synaptic membranes, ensuring efficient neuronal communication [29-31]. Furthermore, LRR proteins have been found to be involved in the regulation of adult nervous system structural plasticity in mammals [28]. In *Drosophila* , transmembrane LRR proteins such as CAPRICIOUS (CAPS) and TARTAN (TRN) are involved in regulating axon and dendrite targeting during the development of neuromuscular, olfactory and visual systems [28]. Both *caps* and *trn* amorphic alleles cause lethal phenotypes during embryonic or postembryonic development [32,33].

Since *2mit* resulted generally expressed in the CNS and PNS during embryonic segmentation, it can be hypothesized that 2MIT plays a role similar to that of other LRR transmembrane proteins involved in neuronal development. However, no lethal phenotypes were observed in *2mit* ^*c03963*^ homozygous flies and analogous results were obtained when *2mit* silencing was induced in neuroblasts and neurons during embryogenesis. In addition, *2mit* ^*c03963*^ homozygous adult flies have not shown any evident morphological abnormalities. The *2mit* ^*c03963*^ allele is a hypomorphic variant of the *2mit* gene, since the *PB* transposon insertion has caused ~50% and ~80% *2mit* mRNA decrements in *2mit* ^*c03963*^ homozygous larvae and adults, respectively. The hypothesis that *2mit* plays an essential role during *Drosophila* development cannot be excluded, since *2mit* residual expression in *2mit* ^*c03963*^ homozygous flies might allow normal neurogenesis and/or vitality.

In wild-type flies, *2mit* expression was detected in adult brains, indicating that this gene plays a role during adulthood. In particular, *2mit* mRNA was identified in both the neuronal somata and axonal lobes of the MBs, and at the level of the EB neuronal fibers. The presence of *2mit* mRNA in the MBs is consistent with data reported by Kobayashi and colleagues, who mentioned *2mit* among those genes preferentially expressed in *Drosophila* MBs [14]. In addition, *2mit* mRNA signals in both the MB axonal structures and EB neuronal fibers of wild-type brains seemed to be specific since they were less visible in *2mit* ^*c03963*^ hypomorphic mutants and absent in negative controls. Given the nature of the 2MIT predicted protein, these data might suggest that *2mit* mRNA is subject to neuronal transport and translational controls. Local control of mRNA translation has been demonstrated within dendrites in several organisms, including *Drosophila*, and it is known to mediate long-lasting synaptic plasticity in the mature nervous system [34]. Different studies have recently provided evidence that regulated mRNA transport and translation occur in both developing and mature axons (for a review, see 35).

In the courtship conditioning test, *2mit* ^*c03963*^ homozygous adult flies have shown normal courtship behavior and courtship suppression during training. These data indicate that *2mit* ^*c03963*^ homozygous males are able to perceive females by integrating visual, olfactory and sensory stimuli, fundamental for courtship behavior [36]. However, *2mit* ^*c03963*^ conditioned males were not able to maintain courtship suppression, evaluated within 5-10 min after training, in the presence of a virgin female, suggesting defects in STM retention. STM impairments were related to *2mit* mRNA depletion since the pan-neuronal 2MIT-HA chimeric protein over-expression in a *2mit* ^*c03963*^ background was able to rescue this mutant phenotype. Moreover, STM defects similar to those observed in *2mit* ^*c03963*^ mutant flies have been noted when *2mit* was silenced in the whole CNS with the *elavGal4* driver or using the more brain-restricted lines OK107-, c772-, and MB247*Gal4*, which strongly expressed GAL4 in the MBs. Additional weaker GAL4-positive regions common to OK107-, c772-, and MB247*Gal4* drivers are located in the OLs [21]. Taken together these data restrict the brain regions potentially involved in the *2mit*-mediated STM phenotype to the MBs and OLs.

In 1999, McBride and colleagues demonstrated that early memory phases in courtship conditioning assays are determined by neuronal circuits outside MBs (such as visual structures and antennal lobes), since chemically MB-ablated flies showed normal memory when tested immediately after training under light conditions [37]. Memory impairments appeared later (with memory decrements found at 30 min and no memory at 60 min after training), indicating that STM consolidation of courtship conditioning required the MB activity. In addition, the same study showed that the ALs play a role in STM retention up to 30 min after training [37]. These data are consistent with the general idea that the memory formation process is a multi-step phenomenon, involving different anatomical structures, including the ALs and OLs, with the MBs representing the brain region for the ultimate storage of memory [38,39]. Defects in the immediate recall of memory in courtship conditioning assays have been demonstrated for *dunce* and *ruta* *baga* mutants (e.g. [40]). Both of these genes are involved in the cAMP signaling pathway, which is fundamental in olfactory learning and memory processes occurring in the MBs [41]. Even if both genes are mainly expressed in the MBs, they are also transcribed at low levels in several other brain regions, including OLs and/or ALs [42,43].

Under our experimental conditions, *2mit* was localized mainly at the level of the MBs in wild-type adult brains, and hybridization signals not clearly distinguishable from the background were detected in both ALs and OLs, at least at ZT 0. Although further studies are required in order to better understand the *2mit*’s role in *Drosophila* STM, it is interesting to note that *in silico* analyses have identified in the 2MIT cytosolic portion two specific motifs for PKA and 14-3-3 protein/s, known to be involved in the control of *Drosophila* memory. In particular, PKA is part of the cAMP signaling pathway [44,45] and two LEONARDO (LEO) 14-3-3 protein ζ isoforms have been shown to modulate memory, acting via an additional signaling pathway, which includes SLOWPOKE Ca^2+^-dependent K^+^ -channel and SLOB [46,47]. Expressed in the CNS during embryogenesis and mainly in the MBs and EB in the adult brain, *leo* shows spatial and temporal expression profiles similar to those of the *2mit* gene [47]. Amorphic *leo* alleles cause embryonic lethality, while hypomorphic variants determined STM defects [47]. It is therefore tempting to speculate that 2MIT functions as a transmembrane LRR neuronal receptor, which in the adult fly influences the memory phenotype as part of a signal transduction pathway.

Our investigations concerning the *tim2* and *2mit* relationship in *D. melanogaster* suggest that there is no functional correlation. In fact, *tim2* is an essential gene required for chromosome stability, which in different organisms has been demonstrated to encode a replisome component [48,49]. In the adult fly, *tim2* has been implicated in circadian light entrainment, probably exerting a function different from that required during development [7]. *2mit* might be involved in nervous system development, and we showed that it plays a role in adult STM. In addition, analyses of *tim2* and *2mit* hypomorphic alleles for STM and circadian light synchronization suggest that these two genes do not have overlapping functions in the adult.

Comparative genomic analyses have shown that the organization of *tim2-2mit* host-nested genes is present in *A. mellifera* and *Drosophilidae* species, suggesting that a *2mit* ancestral gene was located within the *tim2* locus before the *Hymenoptera-Diptera* divergence, which occurred ~300 million years ago [50]. The embedded gene relationship was not maintained in *M. sexta* and *B. mori* moths or in *D* *. plexippus* and *H* *. melpomene* butterflies, indicating that some mobilization event/s involving the *2mit* gene region occurred subsequently within the *Lepidoptera* lineage. It is however interesting to note that the *tim2-2mit* host-nested genomic architecture is preserved in all 21 *Drosophila* species. Among *Drosophilidae*, it has been estimated that only 20-34% of the embedded gene relationships is conserved [1,6], and for those cases the presence of some evolutionary constrains might be hypothesized. It has recently been proposed that nested genes have been conserved throughout evolution by cis-acting transcriptional regulatory sequences located within hosting introns [51]. Enhancer sequence conservation among Vertebrates was demonstrated in an intron of the *LPS-responsive beige-like anchor* (*Lbra*) gene, hosting the nested *Mab21l2* gene [52]. *Lbra* and *Mab21l2* are not functionally related, but their host-nested gene organization has been maintained throughout Metazoan genomes, with the exception of some insect species [52]. In the *D. melanogaster tim2* intron 11, a 6 bp binding site recognized by the SNAIL transcription factor [53] was identified in proximity of *c03963 PB* transposon insertion. SNAIL is known to restrict neuroectoderm and neural fate in invaginating mesoderm and to act as a regulator of neurogenesis in both the CNS and PNS during late embryogenesis. It has been hypothesized that SNAIL may act to repress non neural fates [54]. Comparative genomic analysis detected the presence of a SNAIL binding site in the 11^th^ intron of the *Drosophilidae tim2* locus. These data suggest that this neuronal regulatory sequence could represent a constraint that has maintained the *tim2-2mit* host-nested gene association during the evolution of *Drosophila* species.

## Materials and Methods

### *Drosophila* stocks and maintenance

Flies were raised on a standard agar-yeast-sucrose medium at 23°C in 12:12 LD. *w* ^1118^, *sine oculis* ^*1*^ *, dunce* ^*1*^ *, l(3*)*-31Gal4/TM6B, Actin5C-Gal4/CyO, elavGal4/CyO*, OK107*Gal4*, and c232*Gal4* strains were obtained from the Bloomington *Drosophila* Stock Center (http://flystocks.bio.indiana.edu). c772- and MB247*Gal4* lines were received from C. Helfrich-Foerster, University of Wuerzburg (Wuerzburg, Germany) and the 52Y*Gal4* driver from J.D. Armstrong, University of Edinburgh (Edinburgh, Scotland, UK). Insertional strains c*03963*, *f00075*, *f06803* (carrying a *PB* transposon) and *MB03271, MB08132, MB08962, MB08132* (carrying a *MB* element) were obtained from the Exelixis *Drosophila* Stock Collection (drosophila.med.harvard.edu/). In these strains, *PB* or *MB* transposon insertions map in the *tim2* intron 11. In particular, in *MB08132* and *f06803*, insertions localize in the *2mit* 3’ UTR; in *MB08962*, the insertion maps in the unique *2mit* intron; in *c03963, MB03271*, and *f00075* strains upstream of the *2mit* gene. *PBtim2* ^*c06976*^ (*tim2* ^*c06976*^) and *PBtim2* ^*f00297*^ (*tim2* ^*f00297*^) flies, carrying the *PB* transposon in the *tim2* 5’ UTR and intron 8, respectively, were originally obtained from the Exelixis *Drosophila* Stock Collection and were characterized in [7].

### *2mitO* and *2mit* KD construct production and transgenic line generation

A 3453 bp *2mitHA* chimeric construct (*2mitO*), characterized by 3423 bp *2mit* full-length cDNA followed by an in-frame 27 bp HA (haemagglutinin) tag sequence and a stop codon, was generated for *2mit* over-expression studies. Using specific primers (Table S2), *2mitO* cDNA was initially amplified in four 5’–3’ serial fragments of 792, 1056, 1046 and 739 bp in length, which were cloned in a pCR ^®^II-TOPO^®^ vector (Invitrogen) and checked for errors by sequencing. The four fragments were then digested with appropriate restriction enzymes (Table S2), obtaining 5’–3’ 768 bp *Not*I-*BamH*I, 980 bp *BamH*I-*Sal*I, and 964 bp *Sal*I-*Nde*I consecutive *2mit* cDNA fragments and a 736 bp *Nde*I-*Xho*I segment, coding the 3’ *2mit* region followed by 27 bp HA sequence. These segments were sequentially subcloned in a pBluescript® II S/K (+/-) vector (Invitrogen), obtaining the 3453 bp *Not*I-*Xho*I *2mitO* cDNA, which was then transferred into a pUAST vector.

For KD studies, the *UAS-2mit* RNAi construct (*2mit* KD) was generated as in [55], using a 1234 bp fragment of *2mit* cDNA (positions 2756-3989 in NM_142001.2) without off-target effects as predicted by a bioinformatic program of the Vienna Drosophila RNAi Center (VDRC, http://stockcenter.vdrc.at/). The 1234 bp cDNA fragment was amplified with the primers listed in Table S2.

Both *2mitO* and *2mit* KD transgenic lines were obtained by P-element-mediated transformation following standard procedures [56]. Three independent *2mitO* transgenic lines were generated by the *Drosophila* Embryo Injection Service (Transflier, University of Ferrara, Ferrara, Italy): *2mitO* ^*F8*^ [insert on 3^rd^ chromosome (Chr)], *2mitO* ^*M4*^ (insert on 2^nd^ Chr), *2mitO* ^*M14*^ (insert on 2^nd^ Chr). Three independent *2mit* KD transgenic lines were obtained in our laboratory: *2mit* KD^*6*1^. (insert on 3^rd^ Chr), *2mit* KD^16.2^ (insert on 2^nd^ Chr) and *2mit* KD^61.1^ (insert on 2^nd^ Chr). Insert localization along polytene chromosomes was determined by *in situ* hybridization [55].

### Generation of *Drosophila* *lines* for molecular and behavioral analyses

Molecular and behavioral analyses were performed on the *2mit* ^c03963^ strain obtained by out-crossing c*03963* flies into a *w* ^1118^ background for at least eight generations and on *w* ^1118^ controls.

To evaluate the effects of *2mit* over-expression in the *2mit* ^c03963^ homozygous mutant background, using *CyO/Sco*; *MKRS/TM6B* balancing stock, we initially generated the *elavGal4/CyO; 2mit* ^c03963^ strain, and *2mitO M4*/*CyO; 2mit* ^c03963^ and *2mitO M14*/*CyO; 2mit* ^c03963^ lines, carrying *elavGal4* or *2mitO* constructs over a *CyO* balancer and the homozygous *PB c03963* transposon insertion on the 3^rd^ Chr. The *2mitO* ^*F8*^ *, 2mit* ^c03963^ strain, characterized by the presence of both *2mitO* and *PB* c*03963* inserts on the 3^rd^ Chr was generated via genetic recombination in F1 females, obtained by mating homozygous *2mitO* ^*F8*^ and *2mit* ^c03963^ parental lines and crossed to *MKRS/TM6B* balancing stock. Red-eyed *2mitO* ^*F8*^ *, 2mit* ^*+*^ */TM6B* or *2mitO* ^*F8*^ *, 2mit* ^c^ *03963*/*TM6B* F2 flies were singly mated with *w*; *MKRS/TM6B* flies and checked for recombination via PCR, using specific primers to identify the *PB* c*03963* insertion (Table S2). One F3 recombinant *2mitO* ^*F8*^ *, 2mit* ^c03963^ /*TM6B* line was then selected for subsequent studies. The effects of *2mit* over-expression in a *2mit* ^c03963^ homozygous mutant background were evaluated on *elavGal4*>*2mitO; 2mit* ^c03963^ flies and compared to *elavGal4*>*+; 2mit* ^c03963^ and *+*>*2mitO; 2mit* ^c03963^ relative controls, obtained by mating *2mit* ^c03963^ homozygous flies with those bearing the *elavGal4* driver or *2mitO* construct alone in the *2mit* ^c03963^ background.

2 *mit* KD effects were analyzed in *Gal4*-driven *2mit* KD flies compared to *Gal4>+* and *+>2mit* KD flies obtained by mating *w* ^1118^ flies with individuals carrying either a *Gal4* driver or the *2mit* KD construct alone.

Evaluation of *tim2* depletion effects was performed on heterozygous *tim2* ^*-*^ */+* for two different *tim2* ^*-*^ alleles (*tim2* ^*c06976*^and *tim2* ^*f00297*^) obtained by mating *tim2* ^*-*^ */TM6B* flies to *w* ^1118^ flies.

### RNA isolation, QPCR, and Northern blotting

Total RNA was obtained from L3, 3-5 day-old adult heads and dissected brains. 3-5 day-old flies raised in 12:12 LD conditions or after 3 days in DD were sampled at 3 or 4 h intervals. Adult heads were separated from bodies according to [57]. Brains were dissected in PBS, fixed in 4% paraformaldehyde in PBS for 30 min at 4°C and washed three times for 10 min in PBS. Total RNA was extracted from samples using Trizol™ Reagent (Invitrogen) following the manufacturer’s protocol. The cDNA was synthesized from 1 µg of total RNA using SSII Reverse Transcriptase (Invitrogen) and an Oligo(dT)_20_ primer. QPCR reactions were performed in a 10 µl reaction volume, containing 200 nM of specific primers (Table S2), 5 µl GoTaq® qPCR Master Mix (Promega) and ~30 ng of cDNA per sample. QPCR was performed in triplicate and repeated three times on an ABI7500 system (Applied Biosystem), with the following amplification profile: 95°C for 2 min, 40 cycles of two-step amplification (95°C for 25 sec and 60°C for 60 sec), and melting curve (60–90°C with a heating rate of 0.5 °C/10 sec). To evaluate differences in gene expression we chose a relative quantification analysis based on the standard curve method [58]. Levels of expression were compared with those of an endogenous control transcript (*rp49*) that did not appear to be differentially expressed under our experimental conditions.

Northern blotting was carried out as in [59], using a ~1.7 kb 3’ *2mit* probe (3040-4722 positions in NM_142001.2) and *rp49* full-length cDNA (U92431) as standard.

### *In situ* mRNA hybridization and immunohistochemistry

mRNA *in situ* hybridization on embryos was carried out as in [60] with Fluorescein-labelled 980 nt antisense and sense 2mit RNA probes (2060-3039 positions in NM_142001.2). The *2mit* sense probe was used as negative control. Hybridization signals were detected using an alkaline phosphatase-conjugated anti-Fluoroscein antibody (1:2000, Roche) and NBT/BCIP substrates (Roche).

Biotin labeled RNA antisense and sense ~1.7 kb *2mit* RNA probes (3040-4722 in NM_142001.2) were made for mRNA hybridization on whole-mount adult brains using the Biotin RNA Labelling Mix (Roche). The *2mit* sense probe was used as negative control. *In situ* hybridization experiments were performed on 3-5 day-old adult brains collected at ZT 0, in 12:12 LD conditions. Sample collection, tissue fixation, and mRNA *in situ* hybridization procedures were performed as described in [7]. Samples were hybridized at 65°C overnight with 100 ng probe. Probe detection was performed using TSA^TM^ Signal Amplification kit (PerkinElmer) following the manufacturer’s instructions, incubating samples at 4°C overnight in Streptavidin (1:100) and 3 h in tyramide solution (TSA^TM^ Cyanine 3 System). To visualize the presence of both *2mit* mRNA and 2MIT-HA chimeric protein in OK107*Gal4*>*2mitO* adult brains, *in situ* hybridization protocols did not include treatments with Proteinase K [7]. After *in situ* procedures, brains have been incubated at 4°C for 3 days with a rabbit anti-HA antibody (1:600; Sigma) and at 4°C overnight with a goat anti-rabbit IgG-Alexa 488 (1:250; Invitrogen). Samples were mounted in Vectashield H-1000 (Vector Laboratories) and microscopic analyses were performed using a Leica TCS SP5 II confocal microscope (Leica Microsystems). At least 10 brains for each genotype were analyzed. For each brain, optical sections (Z-series) were taken at 0.5 µm intervals. Post-acquisition analysis and Z-stack construction were performed with Fiji, an open source image processing package based on ImageJ (http://fiji.sc/wiki/index.php).

### Western Blotting

Adult fly whole-bodies were homogenized in extraction buffer as in [61]. After 2 min of sonication, β-dodecyl maltopyranoside detergent was added (1% final concentration). Samples were placed on a rotating wheel for 1 h at 4°C to allow membrane solubilization. According to [62], 0.2 volumes of 5% sodium deoxycholate were added and samples were incubated 10 min on ice. Lysates were centrifuged twice (2800g, 4°C); supernatants were diluted in LDS loading buffer (Invitrogen) and DTT 1 M (0.73 X final concentration; Sigma) and placed 10 min at 70°C. SDS-PAGE was performed using 3-8% NuPAGE® Tris-Acetate pre-cast gel (Invitrogen). After blotting, nitro-cellulose membranes (Trans-Blot Transfer Medium; Bio-Rad) were incubated with a rabbit anti-HA antibody (1:1000; Sigma) and a goat anti-rabbit IgG-HRP (1:1000; Santa Cruz Biotecnology Inc.). Positive immunoreactivity was visualized using the ECL detection system.

### Evaluation of *ls/s-tim1* haplotypes

The single 294 Guanosine insertion/deletion polymorphism existing in the *D. melanogaster tim1* gene was determined as in [24] using the Amplification of Refractory Mutations System (ARMS) PCR method on single flies. Primers are listed in Table S2.

### Egg-to-adult viability

For each genotype, vitality test experiments were performed collecting ~100-300 embryos and counting developing L3, pupae, and adult flies.

### Phototactic behavior

Phototactic behavior was tested in a maze consisting of a series of Y and T tubes (4 mm external diameter) interconnected by transparent 1.5 cm long plastic tubes similar to those proposed to test geotaxis behavior by [63]. Each maze had a single entrance at one side and eight terminal ends at the other. The eight terminal ends were closed with funnel traps, as described in [63]. Mazes were placed horizontally in a box, with black internal walls and a white LED light (130 lux) placed in a corner, corresponding to one of the maze terminal edges. Before testing, flies were maintained in dark conditions. Analyses were performed at the ZT 0-2 time interval on 3-5 day-old males. During each trial, 10 flies were placed at the entrance of the maze and after 1 h each fly received a score from 0 to 7, reflecting the number of positive choices towards the light source.

### Locomotor activity analyses

3–5 day-old flies were individually transferred into transparent tubes (1.5 cm diameter and 4.5 cm length). Tubes were placed inside a black box under white light (141 lux) and locomotor activity was recorded during a 10 min period for each fly. Data were processed by AnyMaze software (Stoelting, Wood Dale, IL, USA). The following parameters were evaluated: total distance moved (m; sum of the distance between each point in the track); average speed (mm/sec); total number of immobility episodes (number of transitions from mobility to an immobile state exceeding 2 sec); total immobility time (sec; sum of duration of each immobility episode). Recordings were performed from ZT 1 to ZT 7.

### Circadian locomotor analyses

Locomotor activity was recorded using the *Drosophila* Activity Monitoring System© (DAMSystem, Trikinetics Inc., Waltham, MA, USA). PRCs were obtained subjecting flies to 20 min light pulses (400 lux) delivered at ZTs 13, 15, 17, 19, 21, and 23. Phase changes were calculated as described in [64]. Positive and negative values represent advanced and delayed phase shift responses, respectively.

### Memory analyses

Courtship conditioning assays were performed as described in [15,16]. Conditioned male flies and sham controls for each genotype were analyzed. Briefly, 4 day-old virgin males, previously kept alone as soon as they eclosed, were individually placed with a 4-5 day-old wild-type *OR-R* mated female for a 1 h training period (conditioned males) or were kept alone (sham controls) in a mating chamber (8 mm diameter and 3 mm high). Males were individually transferred to a new mating chamber and, within 5-10 min, were tested for 10 min with a CO_2_-anesthetized wild-type *OR-R* virgin female, collected that day. Male courtship activities towards virgin or mated females were videotaped under uniform white light (141 lux). The first and last 10 min of the 1 h training period and the 10 min test were inspected to record male courtship behavior. Males which copulated during the training period or courted less than 1 min during the first 10 min of training period were excluded from analyses. If males copulated during the test period or an anesthetized virgin awakened from anesthesia, the observation period was concluded. A courtship index (CI), defined as the amount of time a male spent courting during the 10 min test period, was calculated for each conditioned and sham male. The training index (TI), defined as the ratio between CIs in the final 10 min (CI_f_) and initial 10 min (CI_i_) of the training period, was calculated [16].

### Statistical analyses

All molecular and behavioral data, except for those regarding memory analyses, were analyzed by parametric one- or two-way analysis of variance (ANOVA) and Neuman-Keuls *post hoc* test. CI data did not approximate normal distributions, evaluated with Lilliefors (Kolmogorov-Smirnov) and Shapiro-Wilk tests, even after arcsine, arcsine squared, or arcsine square root transformation. Therefore, they were non-parametrically analyzed using the Mann-Whitney U test for pair-wise comparisons. Analyses were performed using the Statistica 5.0 package (Statsoft Inc.).

### Bioinformatic tools

Sequence accession and annotation were performed through the FlyBase web platform (release FB2013_03; http://flybase.org; 12 Genomes Consortium 2007, http://rana.lbl.gov/drosophila/; modENCODE project, https://www.hgsc.bcm.edu/content/drosophila-modencode-project) or by the EnsemblMetazoa database (http://metazoa.ensembl.org/index.html). *D. melanogaster 2mit* nucleotide and amino acid sequences were compared with the non-redundant sequences available at the NCBI using BLAST and tBLASTN algorithms. The identified contigs were then analyzed with the Augustus gene prediction tool [25].

A multiple protein sequence alignment was obtained by using the MAFFT program (http://mafft.cbrc.jp/alignment/server/) and subjected to phylogenetic analysis by Maximum Likelihood method, via MEGA 5 software [65]. Genetic distances were calculated using the Jones–Taylor–Thornton algorithm [66] and statistical support for nodes on the tree was evaluated using bootstrapping (500 iteration cycles) [67]. Searching for transcription factor binding sites around *c03963* transposon mapping region was performed using the ConSite tool [68].

### Sequence feature analysis

2MIT protein sequence (SwissProt accession ID: Q9VFY9) annotation was obtained from the Annie server (http://annie.bii.a-star.edu.sg), which integrates the prediction from several computational tools, such as CAST and SEG for low complexity region detection and SAPS for the analysis of amino acid composition. The transmembrane topology prediction was derived from the consensus of tools provided by Annie: HMMTOP, PHOBIUS, and TMHMM. We employed an integrative bioinformatic approach combining sequence and domain database searches with the consensus from predictions of protein structural features. The 2MIT sequence was used as a query to scan the domain databases InterPro and Pfam; the secondary structure was predicted using a consensus approach [69]. Prediction of intrinsic disorder and the presence of signal peptides was assessed using ESpritz [70] and SignalP [71], respectively. The N-terminal sequence of 2MIT was scanned for the presence of repeated units using a combination of different classes of repeat prediction methods: RADAR [72], TRUST [73], and Repetita [74]. The predicted repeats were manually aligned in order to calculate the consensus pattern which defines the repeats in the 2MIT protein and to identify further mis-predictions [75]. The C-terminus was scanned for functional linear motifs using the ELM server [26].

### Alignment construction

The structural template for the 2MIT LRR domain was found using MANIFOLD based on sequence and secondary structure. Initial alignments were generated through systematic parameter variation from an ensemble of similar alternatives. Given the problematic nature of repeated sequences, the best initial alignment was used as a starting point only. Manual refinement was performed using knowledge obtained from the structural alignment of the different predicted repeats. Knowledge of key residues and secondary structure was used to anchor the aligned repeats.

### Molecular modeling

The model for the 2MIT LRR domain was constructed using the HOMER server (*URL*: http://protein.cribi.unipd.it/), which uses the conserved parts of the structure to generate a raw model, which is then completed by modeling the divergent regions with a fast divide and conquer method [76]. Side chains were placed with SCWRL3 [77] and the energy was evaluated with FRST [78]. The final models were subjected to a short steepest descent energy minimization with GROMACS [79] to remove energy hotspots before calculating the electrostatic surface with APBS [80]. The structure is visualized using PyMOL (DeLano Scientific, URL: http://pymol.sourceforge.net/).

## Supporting Information

Figure S1 **Locomotor activity in *2mit*^*c03963*^ and *w*^1118^ flies.** (A) Total distance. F_3,105_=0.21 p=0.88; (B) Average speed. F_3,105_=0.21 p=0.88; (C) Number of immobility episodes. F_3,105_=0.04 p=0.98; (D) Total immobility time. F_3,105_=0.11 p=0.95. *2mit* ^*-*^ : *2mit* ^*c03963*^ homozygous mutant flies. Data are expressed as mean ± SEM with the number of tested flies indicated above each bar.
(TIF)

Figure S2 ***2mit* mRNA and 2MIT-HA chimeric protein in the MBs of OK107*Gal4>2mitO* flies.** Localization of *2mit* mRNA (red, A) and 2MIT-HA chimeric protein (green, B) in the MBs of an OK107*Gal4>2mitO* ^*F8*^ brain; signals are merged in (C). *2mit* mRNA and 2MIT-HA chimeric protein co-localize in the Kenyon cells (arrow) and axonal lobes. * *2mit* mRNA and 2MIT-HA signals in non-MB cells activated by the OK107*Gal4* driver. Images are ~12 µm Z-projections. The following abbreviations are used: α/ α’: vertical mushroom bodies lobes; β, β’, γ: medial mushroom bodies lobes. Bar in (C) represents 15 µm for (A)-(C).
(TIF)

Figure S3 **Memory formation in c232*Gal4>2mit* KD and 52Y*Gal4>2mit* KD flies.** Courtship Indices in sham (white bars) and conditioned (black bars) males for c232*Gal4>2mit* KD and 52Y*Gal4>2mit* KD (*6.1*; *16.2*; *61.1*) lines and relative controls [c232*Gal4*> +, 52Y*Gal4>+* and +> *2mit* KD (*6.1*; *16.2*; *61.1*)]. Data are expressed as mean ± SEM with the number of tested flies indicated above each bar. The CIs of the sham flies were significantly different from those of the conditioned males in all c232*Gal4*- and 52Y*Gal4>2mit* KD lines and relative controls. The number of asterisks indicates the significance level: *: p < 0.05; **: p < 0.005; ***: p < 0.0001.
(TIF)

File S1 **Multiple alignment of 21 *Drosophilidae*, 4 *Lepidoptera* and *Apis mellifera* 2MIT sequences performed by MAFTT software and manually refined.**
(FASTA)

Table S1 **Phototaxis behavior in *2mit*^*c03963*^ flies.**
(DOCX)

Table S2 **Primers used in amplification and cloning experiments.**
(XLS)
