# Supplementary figures and images for: 2mit, an Intronic Gene of Drosophila melanogaster timeless2, Is Involved in Behavioral Plasticity
Source: PLoS One. 2013 Sep 30;8(9):e76351. doi: 10.1371/journal.pone.0076351 (PMC3786989; doi:10.1371/journal.pone.0076351)

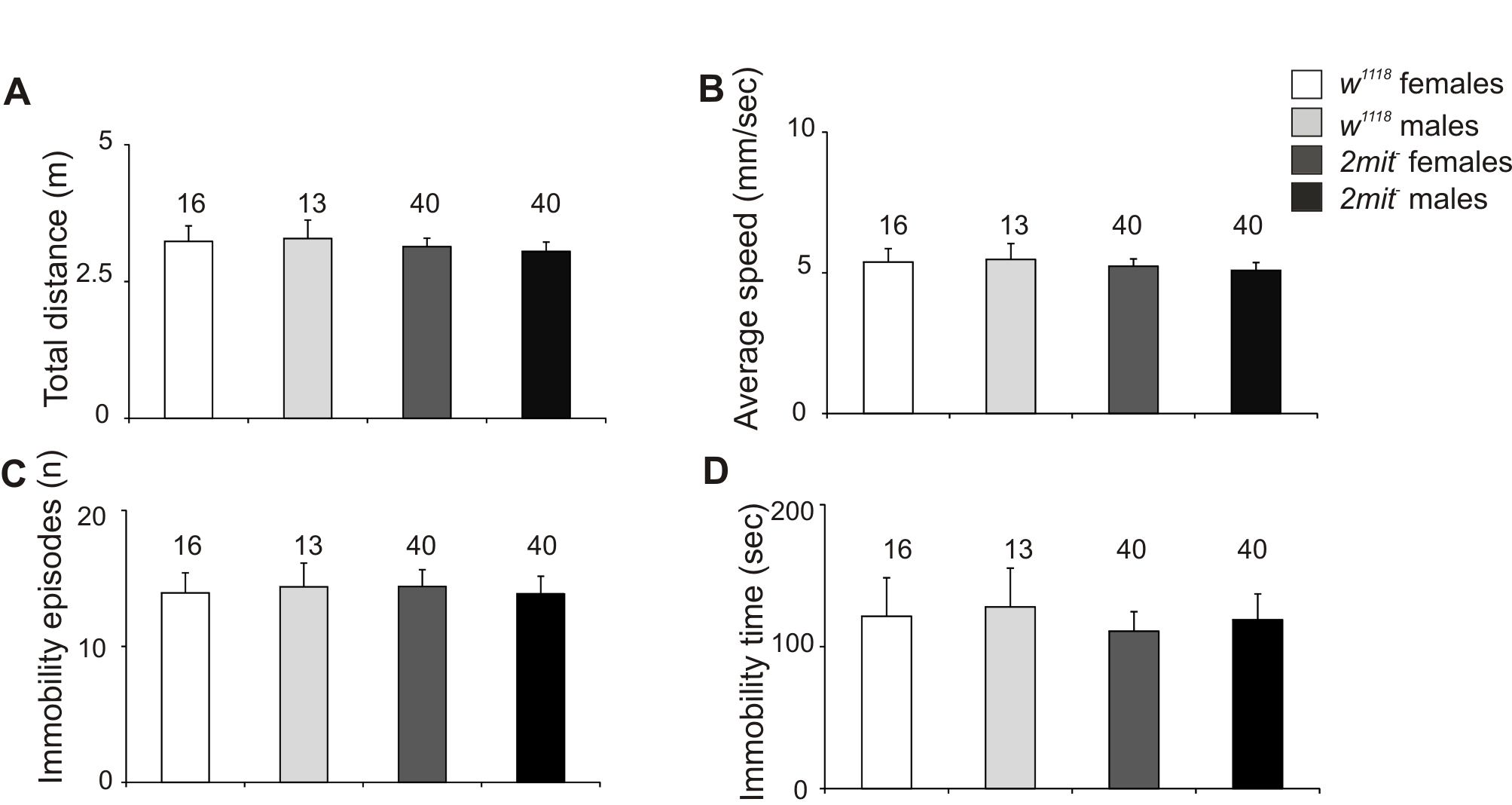

Supplement: Figure S1 — Locomotor activity in 2mitc03963 and w1118 flies. (A) Total distance. F3,105=0.21 p=0.88; (B) Average speed. F3,105=0.21 p=0.88; (C) Number of immobility episodes. F3,105=0.04 p=0.98; (D) Total immobility time. F3,105=0.11 p=0.95. 2mit - : 2mit c03963 homozygous mutant flies. Data are expressed as mean ± SEM with the number of tested flies indicated above each bar. (TIF) [file pone.0076351.s001.tif]

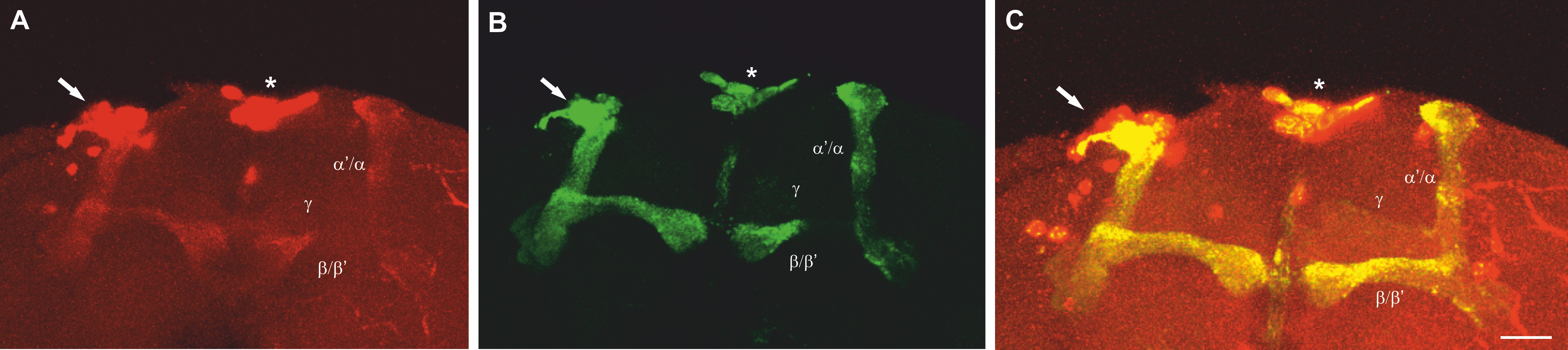

Supplement: Figure S2 — 2mit mRNA and 2MIT-HA chimeric protein in the MBs of OK107Gal4>2mitO flies. Localization of 2mit mRNA (red, A) and 2MIT-HA chimeric protein (green, B) in the MBs of an OK107Gal4>2mitO F8 brain; signals are merged in (C). 2mit mRNA and 2MIT-HA chimeric protein co-localize in the Kenyon cells (arrow) and axonal lobes. * 2mit mRNA and 2MIT-HA signals in non-MB cells activated by the OK107Gal4 driver. Images are ~12 µm Z-projections. The following abbreviations are used: α/ α’: vertical mushroom bodies lobes; β, β’, γ: medial mushroom bodies lobes. Bar in (C) represents 15 µm for (A)-(C). (TIF) [file pone.0076351.s002.tif]

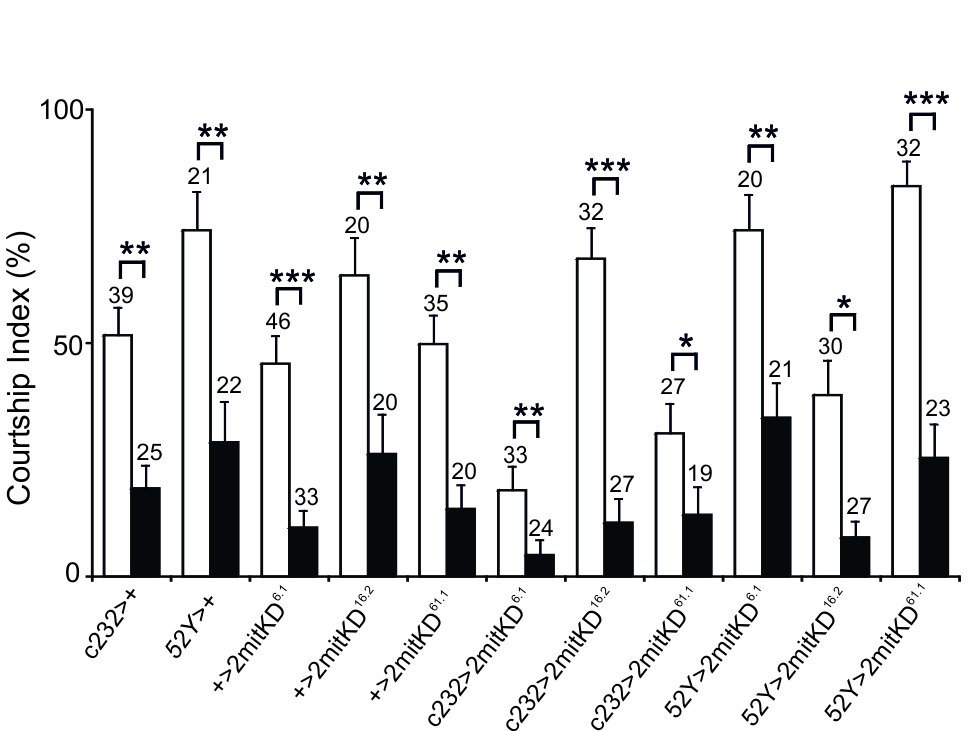

Supplement: Figure S3 — Memory formation in c232Gal4>2mit KD and 52YGal4>2mit KD flies. Courtship Indices in sham (white bars) and conditioned (black bars) males for c232Gal4>2mit KD and 52YGal4>2mit KD (6.1; 16.2; 61.1) lines and relative controls [c232Gal4> +, 52YGal4>+ and +> 2mit KD (6.1; 16.2; 61.1)]. Data are expressed as mean ± SEM with the number of tested flies indicated above each bar. The CIs of the sham flies were significantly different from those of the conditioned males in all c232Gal4- and 52YGal4>2mit KD lines and relative controls. The number of asterisks indicates the significance level: *: p < 0.05; **: p < 0.005; ***: p < 0.0001. (TIF) [file pone.0076351.s003.tif]
