# Supplementary material for: 2mit, an Intronic Gene of Drosophila melanogaster timeless2, Is Involved in Behavioral Plasticity
Source: PLoS One. 2013 Sep 30;8(9):e76351. doi: 10.1371/journal.pone.0076351 (PMC3786989; doi:10.1371/journal.pone.0076351)
Supplement: Table S1 — Phototaxis behavior in 2mitc03963 flies. (DOCX) [file pone.0076351.s005.docx]

**Supplementary Table 1. Phototaxis behavior in** ***2mit^c03963^* flies**

| Genotype | N | Score (mean ± SEM) |
| --- | --- | --- |
| *2mit*^c03963^ | 26 | 5.65 ± 0.26 |
| *OR-R* | 29 | 5.03 ± 0.36 |
| *sine oculis ^1^* | 26 | 1.42 ± 0.31 * |

Phototaxis score (mean ± SEM) for *2mit^c03963^* , *OR-R,* and *sine oculis^1^* adult males. N: number of tested flies. One way ANOVA analysis indicated statistically significant differences among genotypes: F _2, 78_ = 46.28, p<0.0001 significant. Neuman-Keuls *post hoc* test showed not significant differences between *2mit^c03963^* and wild-type *OR-R* flies (p= 0.19, not significant) and significant differences between visually blind *sine oculis ^1^* with both *2mit^c03963^* and wild-type *OR-R* flies (*: p<0.0001 for both comparisons).
